# Capacitance‐Assisted Sustainable Electrochemical Carbon Dioxide Mineralisation

**DOI:** 10.1002/cssc.201702087

**Published:** 2017-12-12

**Authors:** Katie J. Lamb, Mark R. Dowsett, Konstantinos Chatzipanagis, Zhan Wei Scullion, Roland Kröger, James D. Lee, Pedro M. Aguiar, Michael North, Alison Parkin

**Affiliations:** ^1^ Department of Chemistry University of York York YO10 5DD UK; ^2^ Department of Physics University of York York YO10 5DD UK

**Keywords:** aluminium, co_2_ capture, electrochemistry, iron, mineralisation

## Abstract

An electrochemical cell comprising a novel dual‐component graphite and Earth‐crust abundant metal anode, a hydrogen producing cathode and an aqueous sodium chloride electrolyte was constructed and used for carbon dioxide mineralisation. Under an atmosphere of 5 % carbon dioxide in nitrogen, the cell exhibited both capacitive and oxidative electrochemistry at the anode. The graphite acted as a supercapacitive reagent concentrator, pumping carbon dioxide into aqueous solution as hydrogen carbonate. Simultaneous oxidation of the anodic metal generated cations, which reacted with the hydrogen carbonate to give mineralised carbon dioxide. Whilst conventional electrochemical carbon dioxide reduction requires hydrogen, this cell generates hydrogen at the cathode. Carbon capture can be achieved in a highly sustainable manner using scrap metal within the anode, seawater as the electrolyte, an industrially relevant gas stream and a solar panel as an effective zero‐carbon energy source.

## Introduction

The only carbon capture technology that has been implemented on a large scale is amine‐based carbon dioxide capture,^1^, ^2^ which consumes 0.2–0.5 megawatt‐hours per metric tonne of carbon dioxide (MWh t${}_{\mathrm{CO}{}_{2}}$ ^−1^) removed and pressurized to 150 bar.^3^ Difficulties in implementing amine scrubbing technology arise from challenges in storing the pure carbon dioxide gas stream generated and decomposition of the amine capture agent, which generates toxic by‐products.^4^ A number of other technologies have been proposed to separate carbon dioxide from other gases including: oxyfuel combustion, use of solid adsorbants, chemical looping, use of membranes and cryogenic distillation, but to date none of these have been implemented on a large scale.^2^

Electrochemical carbon capture processes have great potential because commercial technology exists to generate electricity from low carbon, sustainable power sources such as solar, tidal or wind energy. Such technology could be applied to capturing carbon dioxide from chemical sources. Cement manufacturing is the largest contributor to global carbon dioxide emissions apart from energy production^5^ and 50 % of this carbon dioxide is an inherent and unavoidable product of the calcination of limestone.^6^ However, research into carbon dioxide (photo)electrochemistry^7^, ^8^, ^9^ is dominated by its reduction to methanol,^10^ methane^11^ or other potential fuels.^12^, ^13^, ^14^, ^15^ Although these processes have a large potential market, they all require large quantities of hydrogen. Non‐reductive carbon dioxide electrochemistry is generally restricted to either the production of niche products such as cyclic carbonates,^16^ oxazolidinones^17^ and α‐hydroxyacids^18^ or to electrochemical mineralisation.^19^

Despite the appeal of trapping carbon dioxide in the form of an inert solid, previous electrochemical mineralisation technologies have been limited by their requirement for expensive ion‐selective membranes that ensure that the concentration of carbonate is high enough to sustain rapid precipitate formation.^20^, ^21^, ^22^ However, it has been shown that carbon electrodes can be used to reversibly uptake carbon dioxide from the gas phase into aqueous sodium chloride solution through supercapacitive swing adsorption.^23^, ^24^ This suggests that this technology could be adapted as a “reagent concentrator” component within an unprecedented dual‐material anode capable of performing irreversible carbon mineralisation through the combination of both electrocapacitive carbon capture and sacrificial metal oxidation. The only previous reports of “bi‐material” electrodes comprise two different chargeable materials, rather than a sacrificial redox‐active component.^25^ Hence, here, we report the design of novel dual‐material, multi‐action electrodes and their use in a sustainable carbon mineralisation technology that operates using aqueous sodium chloride as the electrolyte, without the requirement for base.

## Results and Discussion

### Low‐energy carbon mineralisation

**Figure 1:**
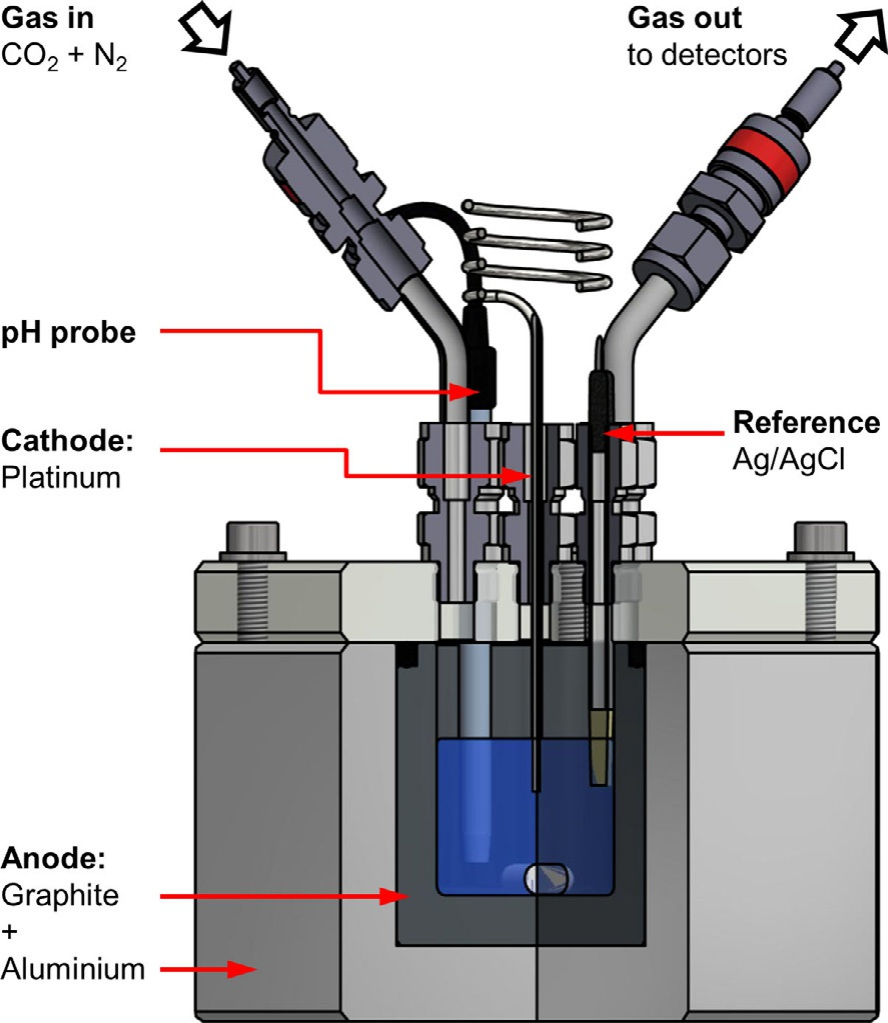
Aluminium–graphite anode electrochemical cell used for carbon dioxide capture. For other diagrams see Figure S1.

The cell shown in Figure 1 was constructed to comprise a graphite‐lined aluminium anode vessel and a platinum wire cathode. The porosity of the graphite liner ensured that there was surface contact between the electrolyte, aluminium and graphite. To further facilitate solution access to the aluminium portion of the anode, eight holes of diameter 3.2 mm were drilled through the base of the graphite (Figure S1). To enable careful monitoring of the solution electrochemistry, the electrochemical cell was fitted with a pH electrode and a Ag/AgCl reference electrode, against which the absolute voltage of the anode and the cathode could be monitored.

**Figure 2:**
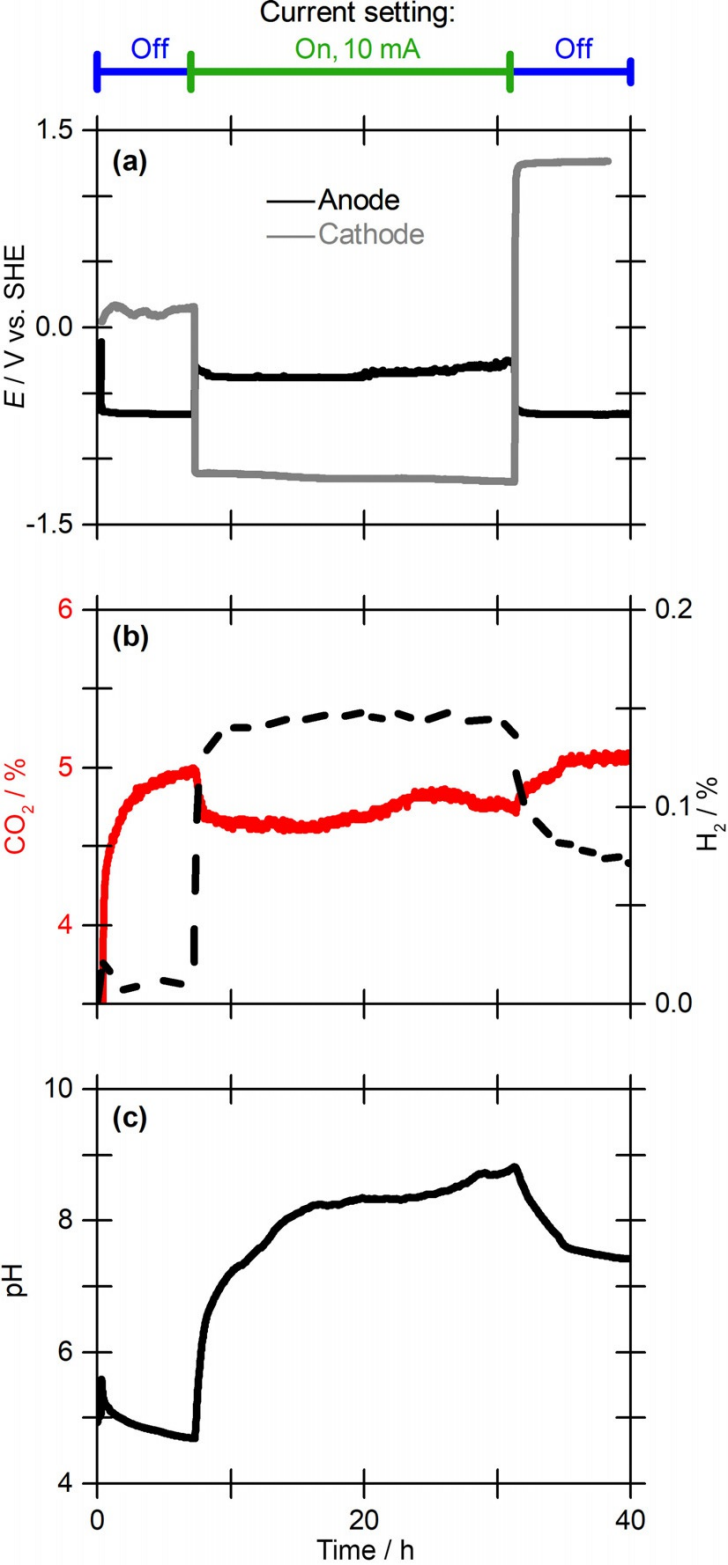
Carbon dioxide fixation using a 10 mA current over 24 h with 1 m aqueous NaCl as electrolyte in the dual‐material aluminium–graphite anode cell. a) Voltage of the aluminium–graphite anode (black line) and platinum cathode (grey line); b) carbon dioxide (red line, left *y*‐axis) and hydrogen (dashed black line, right *y*‐axis) content in the exit gas stream; c) solution pH.

To demonstrate that the cell would capture carbon dioxide, 1 m aqueous sodium chloride was used as electrolyte and a gas stream comprising 5 % carbon dioxide and 95 % nitrogen was passed across the surface of the electrolyte at a flow rate of 14 mL min^−1^, whilst the solution was agitated by a stirrer bar. This gas composition was chosen to mimic the carbon dioxide concentration typically present in waste carbon dioxide sources such as gas‐turbine power station flue gas.^26^ Figure 2 shows the results obtained from a 38 h experiment. During the first 7 h, the cell was at open circuit (the electrodes were held at a potential such that no current flows between them) and the carbon dioxide level in solution reached equilibrium with the gas‐phase carbon dioxide level, as indicated by both the gas trace and the stabilisation of the pH. A constant current of 10 mA was then applied to the cell for 24 h, resulting in an average potential difference of 0.8 V between the dual‐material anode and the platinum cathode (Figure 2 a). Upon application of the current, there was a corresponding drop in the percentage of carbon dioxide in the outlet gas stream (Figure 2 b), showing that the cell performs carbon capture.

The cathode voltage of approximately −1.0 V versus SHE (standard hydrogen electrode) is sufficient for hydrogen production^27^ and this is detected in the outlet gas stream (Figure 2 b). The anode voltage (ca. −0.2 V vs. SHE) is too low to enable unwanted oxygen or chlorine production.^27^ During the 10 mA current stage, there was an increase in the solution pH, which levels off at a value of approximately 9 (Figure 2 c). When, at a time point of 31 h, the cell current is turned off, the carbon dioxide uptake stops and the electrolyte pH level drops.

**Figure 3:**
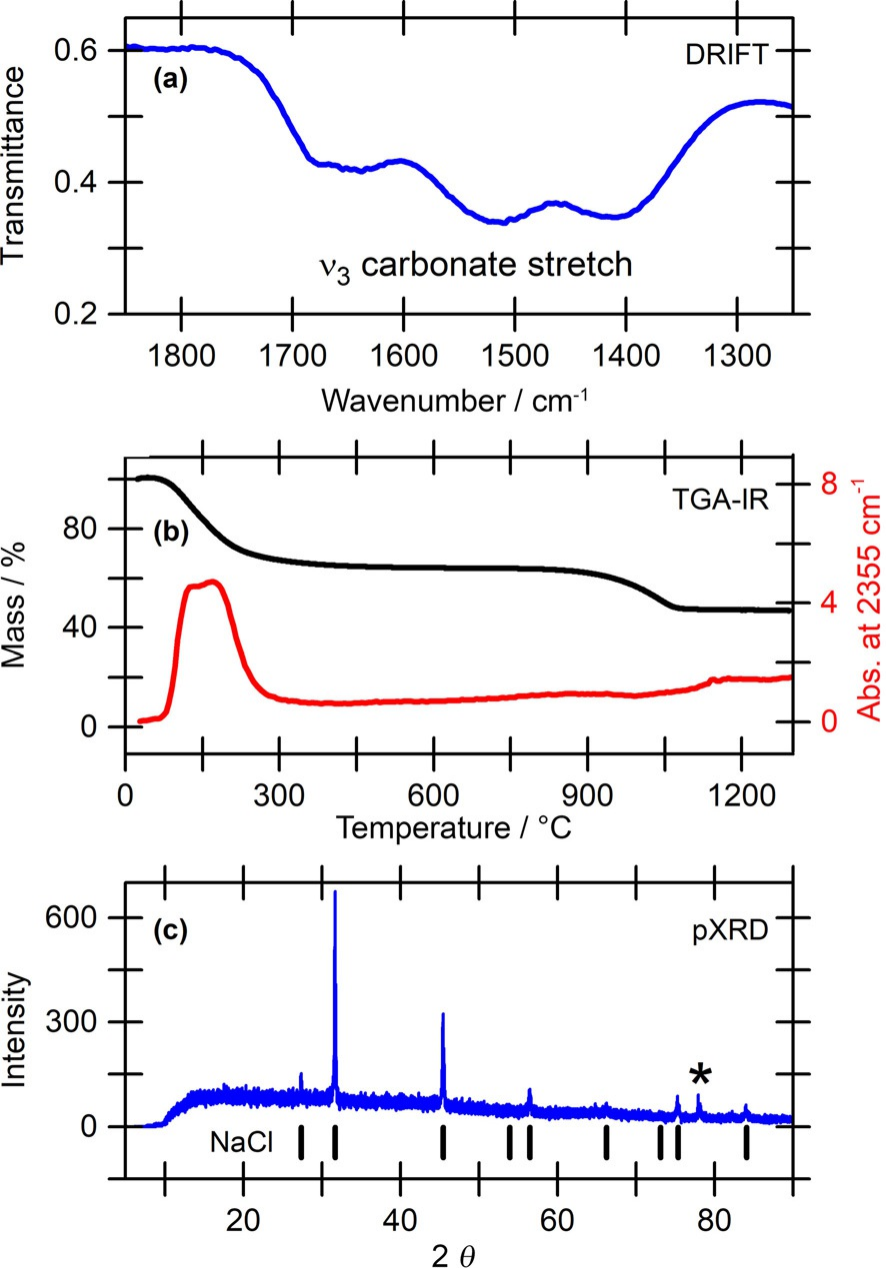
Analysis of the precipitate isolated after carbon capture. a) DRIFTS; b) TGA–IR analysis, black line indicates mass loss as a function of temperature (left *y*‐axis) and red line indicates gas‐phase carbon dioxide detection (right *y*‐axis); c) powder XRD data, the broad featureless signal is due to disordered material, whereas the sharp features are assigned to crystalline sodium chloride (reference values shown by vertical black lines), * signal from metal (aluminium) sample holder.

At the end of 24 h 10 mA applied current experiments, the cell was found to contain 0.30–0.45 g of a light grey precipitate (dried mass). Diffuse reflectance infrared Fourier transform spectroscopy (DRIFTS) of the solid indicated the presence of carbonate through peaks at 1513 and 1407 cm^−1^ (Figure 3 a).^28^ Raman spectroscopy also showed absorbance peaks in the region characteristic of carbonate‐containing materials (Figure S2).^29^, ^30^

Thermogravimetric analysis–IR (TGA–IR) showed that carbon dioxide was released from the precipitate when it was heated to 100–200 °C, which is similar to the lower temperature of carbon dioxide release from commercial basic aluminium carbonate (Al(CO_3_)(HCO_3_), Figures 3 b and S3). TGA–IR analysis of various sodium carbonate and hydrogen carbonate salts showed that loss of carbon dioxide from hydrogen carbonates occurs below 200 °C, whereas loss of carbon dioxide from carbonates only occurs above 800 °C (Figure S4). The mass loss at 900 °C does not involve release of CO_2_ and corresponds to loss of sodium chloride (Figure S3). Comparison with calibration data from a calcium carbonate standard quantified that on average, 0.9±0.2 mmol carbon dioxide is captured by the precipitate (Figure S5, Table S1).

^13^C{^1^H} magic angle spinning (MAS) and cross‐polarisation‐MAS solid‐state NMR experiments also showed a broad signal at *δ*=163±1 ppm that is consistent with that observed for commercial basic aluminium carbonate [(Al(CO_3_)(HCO_3_)] (Figure S6, Table S2). SEM imaging showed crystallites that were present on the surface of the bulk material and these varied in size from 0.8 to 1.3 μm (Figure S7). Powder X‐ray diffraction (XRD) identified this crystalline phase as sodium chloride (Figure 3 c), thus explaining the higher temperature mass loss in Figure 3 b and confirmed that the bulk material is an amorphous phase. The sodium chloride crystallites could be removed by careful washing with cold water and powder XRD on the remaining material showed it to be completely amorphous (Figure S8).

^23^Na MAS NMR spectroscopy (Figure S9–S14, Table S3) confirmed the presence of sodium chloride (*δ*=7 ppm) and revealed a minor (<1 %) secondary sodium environment (*δ*=1±2 ppm, *P*_Q_=1.5±0.4 MHz) consistent with a carbonate‐type phase. Inductively coupled plasma mass spectrometry (ICP‐MS) quantified the total sodium and aluminium content of the solid as summarised in Table 1. Together, these analytical results indicate the formation of an amorphous aluminium and hydrogen carbonate‐containing material along with sodium chloride crystallites. The structure of the amorphous component is discussed in more detail later herein.

**Table 1:** Quantification of carbon, sodium and aluminium in the solid formed and of the carbon in the electrolyte post‐carbon capture.^[a]^

Run	Solid analysis			Solution analysis	
	C [mmol]^[c]^	Na [mmol]^[d]^	Al [mmol]^[d]^	C [mmol]^[e]^	C total [mmol]^[b]^
1	0.6±0.1	7.2±0.2	4.1±0.4	1.3±0.1	1.9±0.1
2	1.0±0.2	6.2±0.2	3.9±0.2	1.7±0.1	2.7±0.2
3	1.0±0.1	3.4±0.2	3.3±0.3	1.9±0.1	2.9±0.1
4	1.1±0.1	8.4±0.5	4.1±0.4	2.6±0.1	3.7±0.1

[a] Each solid and solution analysis was repeated three times and the values quoted represent mean±standard deviation. Each run corresponds to analysis of the solid isolated from a different electrochemical experiment. [b] The carbon total is calculated from summing the solid and solution analysis values and the error is calculated through error propagation of the standard deviation of the contributing values. [c] Calculated from the TGA–IR analysis. [d] Calculated by ICP‐MS analysis. [e] Obtained by titration analysis of the electrolyte post‐carbon capture.

In addition to the carbon dioxide captured within the precipitate, carbon dioxide was also captured within the electrolyte solution as sodium hydrogen carbonate. Based on quantification by Vogel's titration method,^31^ the average hydrogen carbonate content in the electrolyte was 1.9±0.5 mmol (Table 1), whereas negligible amounts of carbonate were detected. Summing together the carbon captured in solid and solution phases, an average total carbon capture of 2.8±0.9 mmol is obtained (Table 1). Over the 24 h, 10 mA period, a total of 691 J of energy was consumed, equating to an energy cost of 247 kJ mol^−1^ of captured carbon dioxide, or 1.6 MWh t${}_{\mathrm{CO}{}_{2}}$ ^−1^. If instead of the average total carbon captured, the maximum and minimum values are used (3.7 and 1.9 mmol), then the energy required is between 187 and 364 kJ mol^−1^ or between 1.2 and 2.4 MWh t${}_{\mathrm{CO}{}_{2}}$ ^−1^. Although these figures are an order of magnitude greater than those reported^3^ for amine‐based carbon capture (0.2–0.5 MWh t${}_{\mathrm{CO}{}_{2}}$ ^−1^), this is not an equal comparison. Amine‐based carbon capture has been optimized over many years and scaled up to pilot plant scale. In contrast, this work was performed at bench scale in a first‐generation electrochemical cell designed for flexibility of operation and to demonstrate the principles. It is highly likely that an order of magnitude improvement in efficiency will be achieved in an optimally designed cell, especially as the energetic value of the hydrogen produced has not been taken into account in the above calculation. In addition, amine‐based carbon capture only captures the carbon dioxide, additional energy will be required to utilize or store it. In contrast, the electrochemical mineralisation developed herein both captures and converts carbon dioxide.

In experiments where a 10 mA current was applied for 36 h rather than 24 h (Figure S15), an average amount of 1.3 mmol carbon was captured in the solid, whereas 3.4 mmol was trapped in solution, showing that both quantities rise with increased experimental time, consistent with the generation of sodium hydroxide at the cathode being essential for the formation of both products. The average energy cost of this carbon fixation was 270 kJ mol^−1^. The increase in this value relative to 24 h experiments can be attributed to the fact that the amount of carbon in the solid did not increase by the same scalar factor as the amount of carbon in solution. This suggests that increasing the aluminium‐to‐graphite surface area may be a necessary improvement in future cell designs, as the graphite pores are more susceptible to becoming blocked with precipitate during longer experiments.

### Electrochemical control experiments

A control experiment performed in the same way as Figure 2 but with no electrical current applied showed no change in the carbon dioxide level reaching the detector (Figure S16), thus demonstrating that carbon capture was not purely due to chemical processes associated with the cell components.

**Figure 4:**
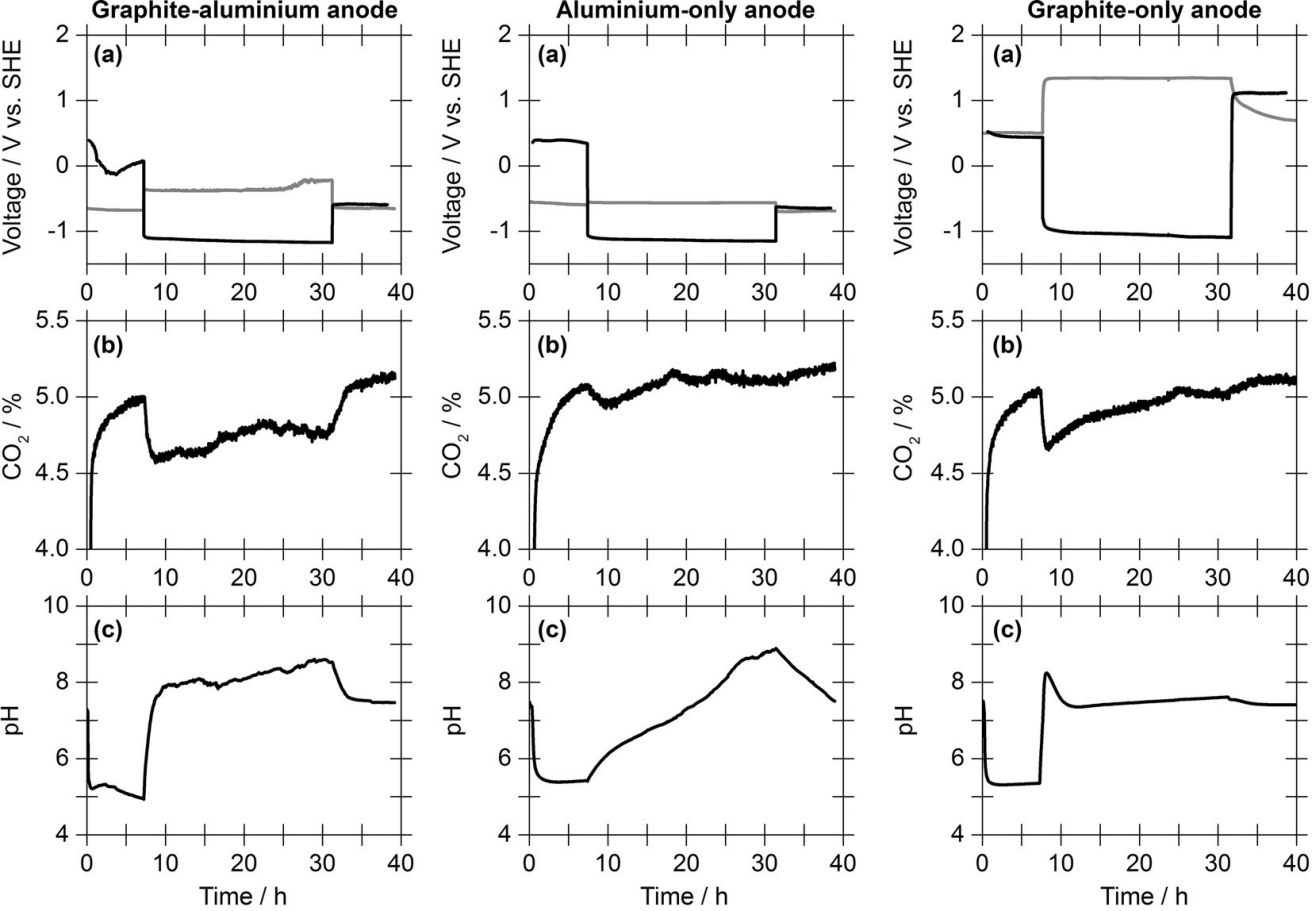
Comparison of the performance of electrochemical cells containing different anodes. a) Anode (grey) and cathode (black) voltages before and after a 10 mA current is applied in a carbon dioxide mineralisation experiment. b) Carbon dioxide content in the exit gas stream. c) Changes in solution pH under carbon dioxide. Other experimental conditions: gas flow of 5 % CO_2_ in N_2_ at 14 mL min^−1^, electrolyte of 1 m aqueous sodium chloride.

In a control experiment in which a 10 mA current was applied for 24 h but carbon dioxide was omitted from the gas stream (100 % nitrogen was used), the electrode potentials responded similarly (Figure S17 vs. Figure 2). This indicates that carbon dioxide is not directly involved in the redox chemistry of the cell. Further electrochemical control experiments were performed using cells containing single‐material anodes of either just aluminium or just graphite (Figure 4). These controls confirmed that the combination of both materials within the anode is necessary to achieve sustained, low‐energy carbon dioxide capture.

When using an aluminium‐only anode, a current of 10 mA generated a cell voltage of approximately 0.6 V and about 0.3 g of solid precipitate was formed. However, only low levels of carbon were detected in this solid (0.33 mmol, approximately 40 % of the amount mineralised in experiments using the mixed material anode). Therefore, the bulk of the material is assigned as hydrated aluminium hydroxide [Al(H_2_O)_3_(OH)_3_] based on consideration of the Pourbaix diagram for aluminium (Figure S18). The carbon‐containing phase of the precipitate is designated as amorphous dawsonite,^32^ which can be formed by reaction of hydrated aluminium hydroxide with carbon dioxide and sodium hydroxide.^33^ Combined with the carbon content in the solution, the total amount of carbon dioxide captured is approximately 60 % lower than the dual‐material anode cell (Table 2, entries 1 and 2). These low levels of captured carbon are also reflected by the trace from the carbon dioxide gas detector and the slow change in solution pH (Figure 4 b). Comparison of entries 1 and 2 of Table 2 shows that the capacitive nature of the mixed graphite–aluminium anode reduces the energy of carbon dioxide capture by 46 % compared to use of an anode composed of just aluminium.

**Table 2:** Quantification of carbon, sodium and aluminium in the solid formed and of the carbon in the electrolyte post‐carbon capture.

Entry	Cathode	Anode	Notes	CO_2_ in solution [mmol]	CO_2_ in solid [mmol]	Total CO_2_ [mmol]	Energy used [J]^[a]^	Energy of capture [kJ mol^−1^ ${}_{\left(\mathrm{CO}{}_{2}\right)}$ ]
1	Pt	C+Al_block_	standard cell	1.90	0.90	2.80	691	247
2	Pt	Al_block_		0.76	0.33	1.09	501	460
3	Pt	C		1.89	–	1.89	2244	1187
4	Pt	C+Al_waste_		1.93	0.78	2.71	618	228
5	Pt	C+Fe_disk_		4.19	0.32	4.51	1097	243
6	Ni	C+Al_waste_		2.12	0.73	2.85	812	285
7	Fe	C+Al_waste_		2.29	0.85	3.14	786	250
8	Ni×11	C+Al_waste_		1.83	0.82	2.65	688	260
9	Pt	C+Al_waste_	seawater electrolyte	0.53	0.34	0.87	711	816
10	Pt	C+Al_block_	1 m LiCl_(aq)_ electrolyte	0.80	0.37	1.17	695	594
11	Pt	C+Al_waste_	solar panel, 3 days^[b]^	0.99	0.25	1.25	201	161

[a] Calculated using average *E*_*c*ell_ values at 10 mA. [b] Carbon dioxide flow constant for 3 days, total time exposed to sunlight approximately 24 hours.

When carbon dioxide was actively bubbled through the solution of the aluminium‐only anode cell through an inlet needle for 24 h with no application of electrical current, a solution level of 1.85 mmol hydrogen carbonate was obtained, which is comparable to the solution carbon levels recorded in electrochemical runs using the dual‐material anode cell (Table 1). This is attributed to an increase in gas‐solution interface compensating for the lack of graphite in the aluminium‐only cell. No solid precipitate was observed in the absence of electrical current.

In graphite‐only anode experiments (Figure 4 c), carbon dioxide uptake was achieved but no precipitate was isolated. This lack of mineralisation is expected based on the absence of a sacrificial metal component at the anode. Thus, all of the captured carbon dioxide (1.9 mmol) is found in the solution phase as hydrogen carbonate (Table 2, entry 3). Relative to the aluminium–graphite experiments, the cell voltage was substantially higher (2.0 vs. 0.8 V) resulting in a far less energy efficient carbon dioxide capture system (1187 vs. 247 kJ mol^−1^, respectively). Such high voltages are indicative of water splitting, with proton reduction and water oxidation occurring at the cathode and anode, respectively. This is consistent with the detection of hydrogen in the outlet gas (Figure 2 b), confirming that hydrogen production is associated with the cathode and not with the aluminium‐containing anode.

### Investigating capacitance

**Figure 5:**
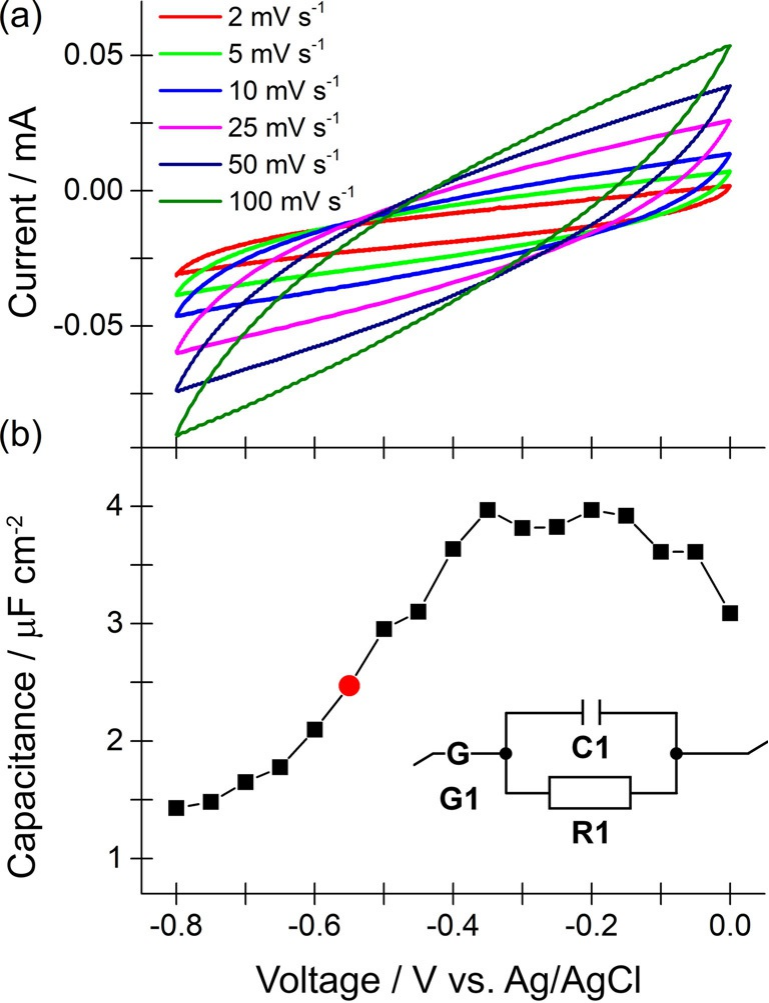
Probing the electrocapacitive properties of the graphite portion of the anode. a) Cyclic voltammograms at different scan rates, as indicated. b) Capacitance as a function of voltage range, modelled against a circuit (inset) to derive capacitance where G1: Gerischer, C1: Capacitor, R1: Resistor. The red circle data point indicates the capacitance at the typical anode voltages used in carbon capture experiments with a dual‐material graphite– aluminium (C+Al) anode. All experiments were conducted using the graphite‐only portion of the anode as the working electrode at 25 °C, in a solution of 1 m sodium chloride and under a headspace of 100 % nitrogen.

Evidence that the graphite portion of the anode plays a capacitive‐only, charging role in the dual‐material anode cell is supported by electrochemical experiments that interrogate a graphite‐only electrode (Figure 5). Cyclic voltammetry experiments (Figure 5 a) showed that graphite is incapable of redox catalysis under anaerobic conditions and across the voltages applied in the carbon capture experiments in Figure 2. Analysis of electrochemical impedance spectroscopy data quantified the capacitance of the graphite electrode as 2.5 μF cm^−2^ at the typical operating voltage for carbon capture (Figure 5 b). This is in close agreement to published values for graphite and validates the assumption that this component of the anode will act as a capacitor.^34^

### Mechanistic studies on the aluminium–graphite anode cell

**Figure 6:**
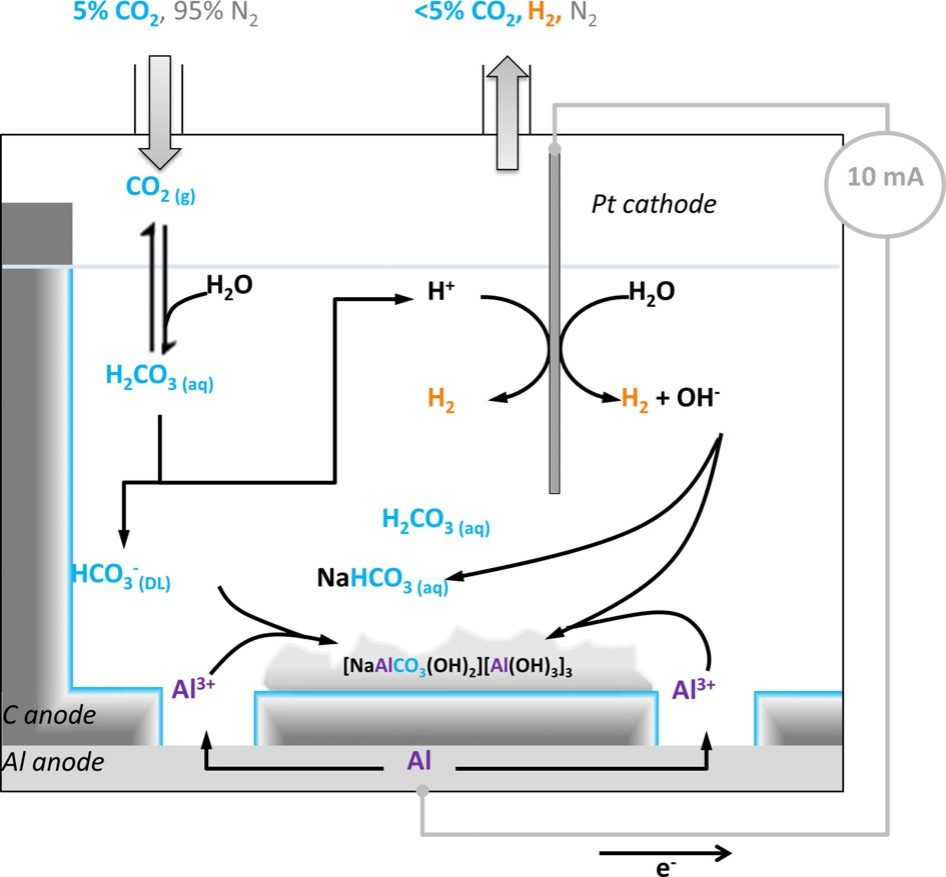
Electrochemical processes leading to carbon dioxide sequestration and mineralization in the aluminium–graphite anode cell. Blue edges indicate an electric double layer.

Figure 6 summarises the reactions occurring in the dual‐material aluminium–graphite anode cell. At the anode, aluminium can be oxidised to form aluminium hydroxide, with proton reduction (resulting in hydrogen and hydroxide formation) catalysed at the platinum cathode giving the net cell reaction A: 2 Al+6 H_2_O→3 H_2_+2 Al(OH)_3_. Alternatively, the oxidised aluminium can act as a carbon capture agent, mineralising carbonic acid, with the net cell reaction B: 2 Al+4 H_2_CO_3_→3 H_2_+2 Al(HCO_3_)(CO_3_). Under the basic cell conditions, the Al(HCO_3_)(CO_3_) reacts with sodium hydroxide to form NaAl(CO_3_)(OH)_2_, a mineral known as dawsonite,^32^ and sodium hydrogen carbonate. As a result of the depletion of the carbonic acid, which occurs in reaction B, the formation of aluminium hydroxide in reaction A and the formation of sodium hydrogen carbonate in the electrolyte, the solution pH increases. The graphite portion of the anode acts as a reagent concentrator, with the supercapacitive swing adsorption action of this electrode component essentially pumping carbon dioxide into solution. Incorporating hydrogen carbonate ions in the double‐layer region of the graphite part of the anode, close to the newly formed Al^3+^, ensures that the ratio of reactions A/B is 7:1 when using a dual‐material graphite–aluminium anode, whereas the ratio of reactions A/B is approximately 21:1 when using an aluminium‐only anode.

Comparing the total charge passed in the 24 h experiments to the total amount of aluminium recovered in the precipitate further supports our conclusion that aluminium oxidation is the major anodic redox process. Assuming that the average 3.9 mmol of aluminium in the solid is in the 3+ oxidation state more than accounts for the 9 mmol of electrons that are passed in the 24 h, 10 mA experiments, that is, the Faradaic efficiency is greater than 100 %.

Given that the standard redox potential for aluminium oxidation is substantially more negative (−1.68 V vs. SHE) than the standard redox potential for proton reduction (0 V vs. SHE), theoretically, the electrochemical cell should generate electrical power rather than consume it.^27^, ^35^ However, the oxide passivation layer that forms on the surface of aluminium always makes oxidation a kinetically inert process, rationalising why an overpotential driving force is required.^36^

### Versatility of cell design

The fundamental design principles of our electrochemical carbon‐mineralisation system are:

1) A dual‐material anode comprising a non‐redox active graphite component, which capacitively generates a region of high carbonate concentration in the immediate vicinity of a sacrificial redox metal that forms an insoluble carbonate.

2) A proton‐reducing cathode.

3) An aqueous electrolyte.

We explored the possibility of varying the cell components to further improve the sustainability of this carbon capture system while working within design constraints 1–3.

#### Anode material

Firstly, the ability to use recycled aluminium at the anode was demonstrated (Figure S19). A graphite cup of the same specifications as in Figure 1, but wrapped in aluminium foil instead of encased in an aluminium block, gave very similar carbon capture compared to the original dual‐material anode cell (2.71 mmol at an energy cost of 228 kJ mol^−1^ vs. 2.91 mmol at 247 kJ mol^−1^, respectively, Table 2, entries 1 and 4).

Next, a mild steel disk (98 wt % iron) was tested as an alternative to using aluminium as the metal component of the dual‐material anode (Figure S20). As expected, based on the proposed mechanism of action, an electrochemical cell of this design also performed carbon capture. However, of the 4.51 mmol carbon retained after the 24 h, 10 mA operation of the cell, only 0.32 mmol was mineralised as solid (Table 2, entry 5). Therefore, although scrap iron could be used instead of aluminium in this carbon capture technology, a lower proportion of isolable mineralised carbon would be achieved. This is attributed to the higher solubility of iron carbonate relative to carbonate containing aluminium salts.^37^ ICP‐MS of the isolated precipitate showed it contained 3.3 % sodium and 41.1 % iron. The solid was amorphous by powder XRD (data not shown).

On average, a potential difference of 1.2 V was measured between the steel–graphite anode and the platinum cathode during the 10 mA current step. This is a larger *E*_cell_ value than the measured using the dual‐material aluminium–graphite anode, which is consistent with the more positive redox potential of iron versus aluminium (−0.04 V vs. SHE and −1.68 V vs. SHE, respectively for a 3 electron processes).^27^, ^38^ The rise in anode voltage that occurs about 24 h into the experiment (Figure S20) is attributed to the accumulation of solid in the cell, which acts to insulate the anode. This indicates that even lower‐energy carbon capture could be achieved through future improvements in cell design.

#### Cathode material

Although platinum is a very efficient and active electrocatalyst for hydrogen production, the rarity and commensurate cost of this material are often quoted as unassailable barriers to any widespread industrial application.^39^ Nickel and iron wires of the same size as the platinum electrode were therefore tested as alternative proton‐reducing cathode materials in cells using dual‐material graphite–aluminium anodes (Table 2, entries 6 and 7, Figures S21 and S22). With these cathodes, the amount of carbon captured in the solid and solution phase of 24 h, 10 mA experiments was shown to be approximately 3 mmol, close to that obtained in analogous experiments using platinum as the cathode material (Table 2, entry 4), though there was an increased energy cost. The electrical power consumption was higher (812 J for nickel, 786 J for iron) because the cathode voltages of the nickel and iron wires (both at −1.19 V vs. SHE) were more negative than when platinum was used (−0.99 V vs. SHE).

With a nickel cathode, the cell voltage could be improved by increasing the length of the wire. Increasing the surface area by a factor of 11 gave a decrease in cell voltage from 0.94 V to 0.80 V, corresponding to a reduction in energy use of 124 J (Table 2, c.f. entries 6 and 8).

#### Electrolyte

To further enhance the green metrics of the technology, UK North Sea water was tested as the electrolyte in the dualmaterial graphite aluminium‐foil anode and platinum cathode cell (Table 2, entry 9; Figure S19). Carbon capture was achieved using this readily available resource, demonstrating the robust scalability of the technology.

**Figure 7:**
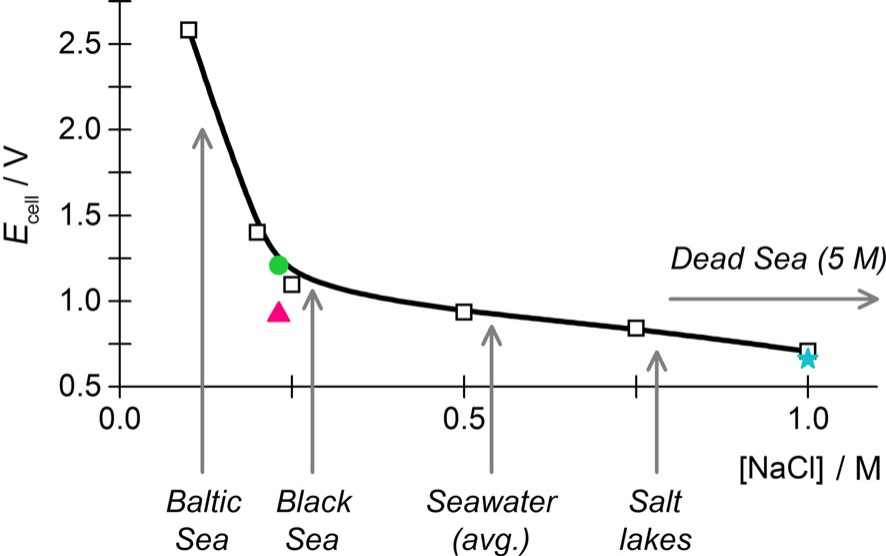
Difference between anode and cathode voltages as a function of sodium chloride concentration. Solid line drawn as a guide to the eye. Whitby seawater sodium chloride concentration was measured by ICP‐MS.

Relative to 1 m NaCl electrolyte experiments, a 24 h, 10 mA carbon‐fixation run using seawater as electrolyte fixed only one third the amount of carbon and required a higher energy input, so that overall, the carbon capture had a cost of 816 kJ mol^−1^. Based on electrochemical tests using aqueous sodium chloride electrolytes of different concentrations (Figure 7), this high‐energy input can be attributed to the low sodium chloride concentration (0.2 m) in North Sea water. A non‐linear relationship between sodium chloride concentration and cell voltage is expected as lower ionic strength will impede dissolution of the oxide layer, which naturally passivates all aluminium electrodes, and also increase solution resistivity.

As highlighted in Figure 7, average global seawater sodium chloride levels are >0.5 m, whereas much higher sodium chloride concentrations occur naturally in salt lakes such as the Dead Sea (up to 5 m NaCl).^40^ Natural sources of water therefore exist which would be expected to support low‐energy carbon capture in our electrochemical cell. To support this reasoning, sodium chloride was added to the North Sea water sample to raise its sodium chloride concentration to 1.0 M. When used as an electrolyte with an aluminium anode and platinum cathode, this electrolyte performed analogously to the use of pure 1 m sodium chloride as electrolyte (compare Figure S23 and Figure 2).

Using a 1 m lithium chloride solution as the electrolyte in the dual‐material graphite–aluminium‐foil anode and platinum cathode cell enabled capture of 1.17 mmol carbon (Table 2, entry 10; Figure S24). The solid formed in this reaction was shown to contain LiAl_2_(OH)_6_Cl and LiAl_2_(OH)_6_Cl⋅H_2_O phases by powder XRD (Figure S25). Such layered hydroxide species were previously proposed as potential carbon‐capture reagents^41^, ^42^ and this method offers a low‐energy and simple route through which they can be generated.

#### Carbon‐free energy source

**Figure 8:**
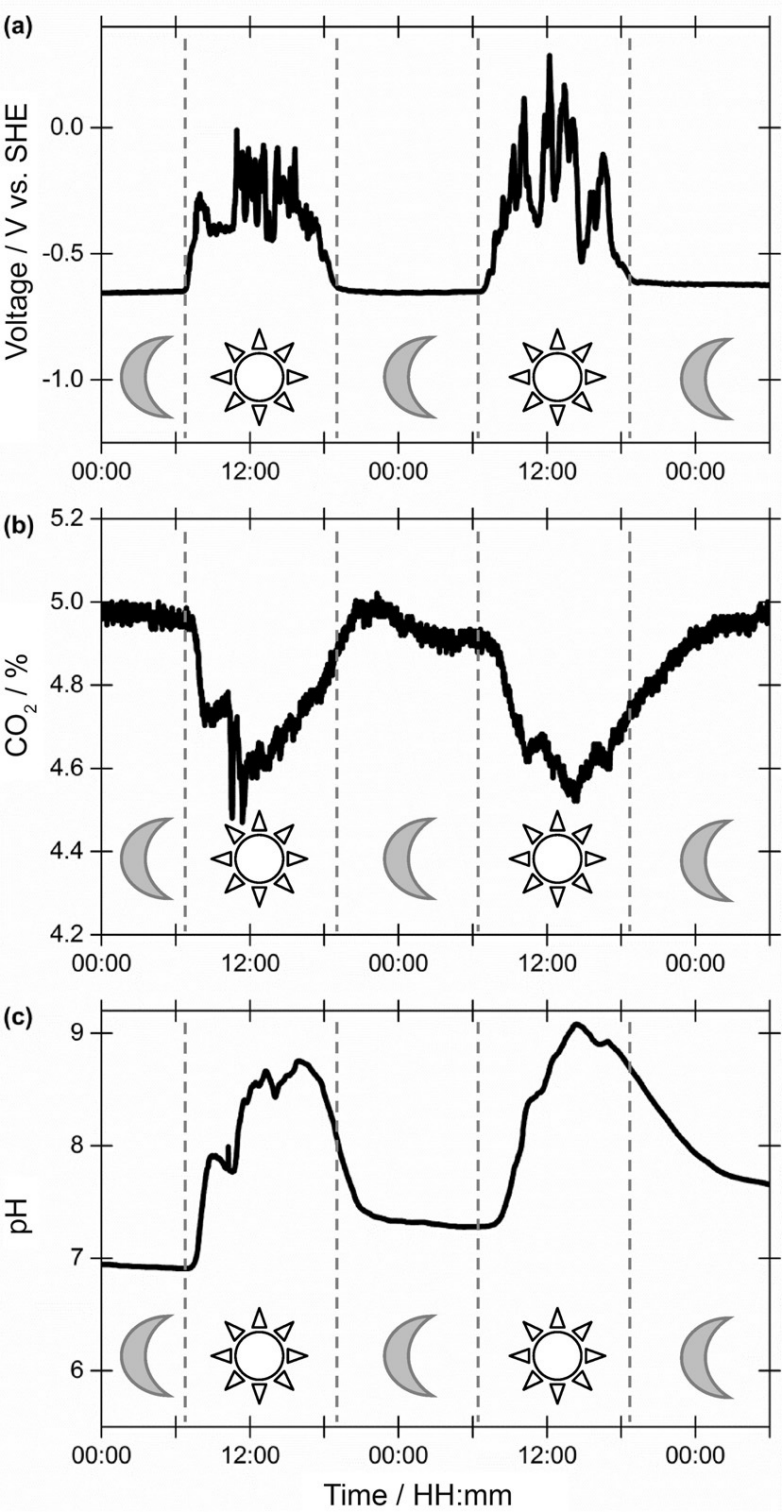
Use of a solar panel to drive carbon dioxide capture in the dual aluminium–graphite anode cell with 1 m aqueous sodium chloride as electrolyte. a) Carbon dioxide level, b) anode voltage and c) solution pH.

Having shown that sustainable materials could be used to build all the electrochemical elements of the cell, a 140 cm^2^ solar panel was used to power the process (Table 2, entry 11), replacing the input power of the potentiostat. Figure 8 shows representative instrument data. An experiment over three consecutive days yielded a low‐energy cost for carbon capture of only 161 kJ mol^−1^ (Table 2, entry 11). We note that the amount of carbon in the solid dropped substantially. This is attributed to the lower current (ca. 3 mA) that flows when the solar cell is used. In control, 24 h applied current experiments where a 3 mA current was passed through the graphite–aluminium block cell instead of 10 mA, only 0.22 mmol CO_2_ was recovered from the solid (data not shown).

### Energetic and scalability analysis

By averaging the data in runs 1–4 of Table 1, 3.9 mmol of aluminium is used to capture 2.8 mmol of carbon dioxide. This corresponds to 1.2 tonnes of carbon dioxide being captured by every tonne of aluminium consumed by the electrochemical process. In 2015, 58 million tonnes of aluminium were manufactured and the recycling rate was 35 %, which corresponds to 20 million tonnes.^43^ If the recycled aluminium was used to electrochemically capture carbon dioxide instead, this would capture 24 million tonnes of carbon dioxide.

Alternatively, if the aluminium that is not currently recycled was used in the electrochemical process, then this 38 million tonnes of aluminium could capture 45 million tonnes of carbon dioxide. These figures compare well with commercialised carbon dioxide utilisation processes (Table 3).^44^ If the electrochemical method was used to create an inorganic carbonate material, it could be the third largest carbon‐dioxide‐utilising chemical process. Production of inorganic carbonates (mostly of calcium, sodium and potassium) is already the second largest use of carbon dioxide and is an expanding market.^45^ Table 4 lists the largest sources of anthropogenic carbon dioxide emissions.^5^ These are dominated by electricity production from the burning of coal or natural gas. Our technology cannot substantially counteract these immense carbon dioxide emission levels, and arguably, neither can any other existing technology. However, as Table 4 shows, essential chemical processes also produce waste carbon dioxide and do so at a scale that could be very significantly offset by the electrochemical carbon dioxide mineralisation process described here.

**Table 3:** Commercial processes which utilise carbon dioxide (data for 2014). ^[44]^

Chemical	Amount produced [10^6^ t year^−1^]	CO_2_ utilised [10^6^ t year^−1^]
urea	155	114
inorganic carbonates	200	50
methanol	50	8
formaldehyde	21	3.5
dimethyl ether	11.4	3
*tert*‐butyl methyl ether	30	1.5

**Table 4:** Global anthropogenic emissions of carbon dioxide (data for 2008).^5^

Source	CO_2_ emissions [10^6^ t year^−1^]	CO_2_ purity [volume %]
electricity production (coal)	14 200	12–15
electricity production (gas)	6320	3–5
cement production	2000	14–33
iron and steel production	1000	15
oil refineries	850	3–13
ethene production	260	12
ammonia production	150	100
natural gas production	50	5–70

Recycling aluminium requires 10 GJ of energy per tonne.^43^ In contrast, taking the average data in Table 1, 3.9 mmol of aluminium captures 2.8 mmol of carbon dioxide at an energetic cost of 691 J. This corresponds to use of 6.6 GJ of energy for every tonne of aluminium used in this way. Thus, electrochemical carbon dioxide mineralisation requires 33 % less energy than conventional aluminium recycling and has the added benefit of capturing 1.2 tonnes of carbon dioxide within the carbonates formed. Taking the worst case scenario, that all the energy required for the electrochemical carbon dioxide mineralisation comes from a coal‐fuelled power station, in the generation of electricity, 500 g of carbon dioxide would be produced for each kWh (or 3.6 MJ) of electrical energy produced.^46^ Thus, generation of 6.6 GJ of energy would produce 0.9 tonne of carbon dioxide. The electrochemical capture and mineralisation would therefore still be a net consumer of 0.3 tonnes of carbon dioxide per tonne of aluminium used. As the percentage of carbon neutral energy in the energy mix increases, the net carbon dioxide consumed will move towards the limiting value of 1.2 tonnes per tonne of aluminium used. The importance of the aluminium component of the anode for this process is shown by performing the same energetic analysis on the graphite‐only cell results. In this case, capture of 1.9 mmol of carbon dioxide required 2.2 kJ of energy so capture of 1 tonne of carbon dioxide would require 27 GJ of energy, which if generated in a coal‐fuelled power station would produce 3.7 tonnes of carbon dioxide, making the graphite‐only cell an overall net emitter of 2.7 tonnes of carbon dioxide for each tonne of carbon dioxide captured.

Using steel as the sacrificial anode in place of aluminium can open a much more expansive resource pool for carbon capture. This cell used 1.1 kJ of energy to capture 4.51 mmol of carbon dioxide bound to 0.80 mmol of iron. This translates to 4.2 tonnes of carbon dioxide captured per tonne of iron. In 2012, the total steel recycled was 1426 million tonnes and the steel not recycled was 195 million tonnes.^47^ If this nonrecycled waste steel was used to perform electrochemical carbon capture and mineralisation, then 822 million tonnes of carbon could be captured. This would essentially negate the effect of oil refineries or many other smaller‐scale processes. Capture of one tonne of carbon dioxide with a sacrificial steel anode component takes 5.5 GJ of energy, which, if generated in a coal‐fired power station would produce 0.8 tonnes of carbon dioxide, leading to a net capture of 0.2 tonnes. The energy of carbon dioxide capture with this cell is very similar to that of current steel recycling at around 5 GJ per tonne.^48^ The energetic analysis will become even more favourable if the value of hydrogen as a by‐product is used to further offset the energy input.

## Conclusions

We demonstrated a completely novel electrochemical process associated with the use of a dual‐material electrode capable of both capacitive charging and providing a source of metal ions. The capacitive charging acts as a reagent concentrator, facilitating a mineralisation reaction between dissolved carbon dioxide and the metal ions. Thus, the carbon dioxide is sequestered as carbonate in the electrolyte solution and as an insoluble aluminium hydroxycarbonate mineral. This is the first time a redox inactive material has been incorporated into the construction of an electrode for the purpose of concentrating a reagent to enhance the efficiency of a reaction.

Carbon mineralisation through the process described can be achieved just by consuming metals (aluminium or iron) that are not geographically concentrated^39^ and available on a substantial “scrap” scale, whilst utilising renewable energy from a solar panel. The sustainability of this process was shown by the use of waste aluminium foil within the dual‐material anode and the use of seawater as the electrolyte.

Given the current annual production rate for aluminium and its recycling rate, this technology could be used to mineralise 20–45 million tonnes of carbon dioxide per annum, which would make it the third largest carbon‐dioxide‐utilising chemical process. Analysis of the energetics of the electrochemical mineralisation shows it is 33 % more energy efficient to use waste aluminium this way rather than to recycle it. A similar analysis for using non‐recycled scrap steel suggests this could capture 822 million tonnes of carbon dioxide, enough to negate the effect of refineries worldwide. Even if the required electrical energy came from a coal‐fuelled power station, the overall process using either aluminium or steel is carbon negative, with more carbon dioxide being mineralised than would be released by the power station.

The technology sequesters carbon dioxide from a dilute gas stream (5 % carbon dioxide in nitrogen) while producing hydrogen as a valuable co‐product, which could also offset the cost of carbon dioxide capture. The platinum cathode hydrogen‐production electrocatalyst, which is not consumed in the reaction, can be replaced by either nickel or iron, further increasing the sustainability and scalability of the process. Future work will focus on optimising the cell design including the surface area and pore structure of the graphite electrode, to optimise gas–liquid and liquid–solid contacts and hence the energy efficiencies. The effect of impurities in the carbon dioxide gas stream will also be investigated. Carbon dioxide purities (and the impurities present) vary enormously depending on the carbon dioxide source.^5^ Power station flue gas is the largest source of waste carbon dioxide and this can be expected to contain nitrogen oxides, sulfur oxides (from coal) and carbon monoxide. Nitrogen and sulfur oxides are routinely removed by scrubbing the flue gas, so the main concern would be carbon monoxide, which may poison a platinum cathode. However, as nickel and iron have been demonstrated to be satisfactory alternative cathode materials, this is unlikely to be a serious problem.

## Experimental Section

### Cell construction

All electrochemical cells were constructed in‐house by the mechanical workshop in the Department of Chemistry at the University of York (Figure 1 and Figure S1). The beaker‐shaped porous graphite portion of the anode electrodes was machined from graphite supplied by OLMEC, grade MCCA, which had a porosity of 14–17 % and a maximum grain size of 0.6 mm (Figure S26). The graphite had a BET surface area of 9.8 mm^2^ g^−1^, a pore volume of 0.02 cm^3^ g^−1^ and was predominantly microporous (Figure S27). The aluminium case was supplied by Alaco (Grade 6082, Temper T6), with an aluminium content of 95–98 %. In the graphite‐only anode cell, a plastic block replaced the aluminium block shown in Figure 1. An aluminium ring was placed on top of the graphite liner, to permit electrical connection between the cell and potentiostat while ensuring that only the carbon portion of the anode came into contact with the solution. The aluminium‐only anode cell resembled that shown in Figure 1, except it was missing the graphite liner. The electrochemical cell used for the seawater and waste aluminium experiments consisted of a plastic block base, into which a graphite liner wrapped in aluminium foil could be inserted (Figure S1). Modifying the anode component to use a mild steel disk involved placing the steel disk underneath the carbon insert and using a mild steel connector to make electrical contact with the aluminium ring as described in the carbon‐only experiments. Each cell base had a matching Perspex lid that sealed onto a recessed O‐ring. Each lid contained five ports; three were used to place a pH probe (Semi‐micro epoxy gel BNC pH electrode, VWR International), platinum, nickel or iron wire electrode (3.5 cm in length, 1.30 mm in diameter) and a Ag/AgCl reference electrode (3 m NaCl, BASi) into the electrolyte and two Swagelok fittings to permit gas‐tight connections. Gas mass‐flow controllers (Aalborg, GC717, 0–10 mL min^−1^ and 0–100 mL min^−1^) were used to control the rates at which carbon dioxide (BOC, >99 %) and N_2_ (BOC, >99 %) passed across the surface of the electrolyte. A Quantek CO_2_ analyser, model 906 and a GC H_2_ detection instrument monitored the outlet gas.

### Electrochemistry experiments

In each experiment, the electrochemical cell was placed on top of a UC152, Stuart magnetic stirrer plate set to a speed setting of 4 and agitation of the electrolyte solution was achieved using a 10×4 mm magnetic stir bar. The stirrer plate temperature was monitored using a thermocouple connected to a data logger; it reached a constant temperature of 35 °C after the first hour of operation.

All electrolyte solutions were made using ultrapure water (Purite, ONDEO) and salts of at least 99 % purity, either NaCl (VWR International) or LiCl (Santa Cruz Biotechnology). All experiments used 60 mL of electrolyte except for the aluminium‐only anode tests (180 mL) and the seawater and waste aluminium tests (90 mL). Approximately 3–4 g of aluminium foil was wrapped around the graphite liner when used as the aluminium source.

A VoltaLab 50 potentiostat with VoltaMaster software was used in experiments with controlled current flow and this monitored the potential of the platinum cathode relative to that of the Ag/AgCl reference electrode. A digital voltmeter with USB data logging (PT‐4000ZC, Digitec) was used to monitor the potential of the anode with respect to the Ag/AgCl reference. All electrochemical potentials reported have been corrected to versus the SHE. When performing experiments with light energy, a Multicomp solar panel was used (MC‐SP0.8‐NF‐GCS, 14 cm tall, 10 cm wide, maximum power 800 mW, maximum voltage 3.85 V, maximum current 0.21 A) and the current and voltage was monitored using Digitek DT‐4000ZC data logging multimeters. The solar panel was placed directly against the glass of a window at a 30–32° N by NE angle. The laboratory is at latitude 53° 57 min 30 s N and experiments were performed from October 29th to 31^st^, 2016.

### Calibration of the Ag/AgCl reference electrode

To determine the conversion factor required to adjust the potentials measured with the Ag/AgCl reference electrode to vs. SHE, cyclic voltammetry measurements were performed in triplicate. These were conducted with 10 mm ferricyanide (K_3_FeCN_6_) in 0.1 m pH 7 phosphate buffer, the Ag/AgCl reference electrode, a glassy carbon working electrode (BASi) and a platinum counter electrode (wire) according to O'Reilly's method (Figure S28).^49^ Analysis was performed with an EmStat^3^ potentiostat (PalmSens) and PSTrace4 software (PalmSens) (Table S4) giving a conversion factor of: *E* [V vs. SHE]=*E* [V vs. Ag/AgCl]+0.194 V.

### pH sensor calibration

The pH probe was calibrated prior to each experiment (Figure S29) using four reference buffer solutions from Fisher Scientific, pH 4 (potassium acid phthalate), 7 (phosphate), 9.2 (borate) and 10 (potassium carbonate).

### Quantification of hydrogen production

The level of H_2_ production was quantified using a commercial hydrogen analyser utilising GC combined with a HgO reduction detector (Ametek TA3000R), designed to detect CO and H_2_ gas. In operation, CO and H_2_ were separated using the gas stream on the GC column and then detected through the reduction of mercuric oxide and the subsequent detection of mercury vapour by UV absorption.^50^ To reduce the concentration to within the instrument range, the outlet flow was diluted by a factor of approximately 500 in ultra‐pure nitrogen. The dilution flow rates were continuously monitored to correct the instrument readings into H_2_ concentrations in the original gas flow. Data from the H_2_ detector was logged and analysed by DAQ factory analysis software.

### Diffuse reflectance infrared Fourier transform spectroscopy (DRIFTS)

Samples were analysed with an Equinox 55 FTIR (Bruker) and OPUS software (Bruker). Samples were mixed with freshly ground and oven‐dried KBr (Fisher‐Scientific, spectroscopy grade) in a 1:10 mass ratio. IR spectra were measured between 4000–500 cm^−1^, with a resolution of 4 cm^−1^ over 128 scans. A background scan was run prior to sample analysis with the same resolution at 256 scans. The mercury cadmium telluride (MCT) detector was cooled using liquid nitrogen prior to each run.

### Raman spectroscopy

Raman spectra were recorded using a 532 nm wavelength laser as the excitation source. A 50× magnification objective lens (0.5× numerical aperture) was used to focus the laser light on a spot size area of approximately 1.5–2 μm in diameter. The total acquisition time was 2 s over 130 repeated scans for each measurement. A 1650×200 pixels size charge‐coupled device detector was used to generate the Raman graphs. The spectral resolution was approximately 1.5 cm^−1^/pixel.

### Thermogravimetric analysis‐infrared spectroscopy (TGA–IR)

TGA–IR analysis was performed with a Netzsch 409 STA TGA twinned with a Bruker Equinox 55 FT‐IR, which used Netzsch Proteus and OPUS software simultaneously. This instrument measures how much solid samples decompose over a given temperature range and also identifies the released gas during the decomposition by in‐line gas IR analysis. Samples were placed in pre‐burnt alumina cups and analysed over the temperature range 25–1300 °C at a ramp rate of 10 °C min^−1^. The TGA was pre‐vacuumed and purged with N_2_ gas three times prior to use. A N_2_ flow rate of 100 mL min^−1^ was run through the TGA during analysis. IR spectra were measured between 4000–550 cm^−1^, with a resolution of 4 cm^−1^ and 64 scans. A background was taken at the same resolution with 128 scans prior to each sample analysis. The transfer line between the TGA and IR was kept at 200 °C during each run. The MCT detector was cooled using liquid nitrogen prior to each run.

### Solid‐state nuclear magnetic resonance (NMR)

All solid‐state NMR spectra were collected using a Bruker AvanceIII HD 400 spectrometer equipped with a 9.4 T wide‐bore magnet and a 4 mm MAS probe. Spectra were acquired under regulated temperature of ca. 298 K (accounting for heating from rotational friction) for most samples. In order to minimise decomposition, spectra of the sodium carbonate decahydrate sample were acquired at ca. 278 K.

^13^C{^1^H} CPMAS experiments employed a 2 ms linearly ramped contact pulse (^1^H channel), spinning rates of 1.25 to 10 kHz, recycle delays of 1–60 seconds, spinal‐64 heteronuclear decoupling (at *ν*_rf_=85 kHz) and are a sum of 48–3000 co‐added transients. For the crystalline model systems with long ^1^H T1s, a flip‐back pulse was utilised to reduce the necessary recycle delay. ^13^C Bloch decay experiments for anhydrous sodium carbonate, sodium sesquicarbonate and solid precipitate samples were acquired using a 1.66 μs pulse (30° tip‐angle) with recycle delays of 30–120 s. Chemical shifts are reported with respect to TMS and were referenced using adamantane (*δ*=29.50 and 38.55 ppm) as an external secondary reference.

^23^Na{^1^H} MAS experiments were acquired using a Bloch‐decay sequence employing a 0.83 μs pulse (at *ν*_rf_=42 kHz), spinning rates of 5 to 14 kHz (10 kHz for most samples), optimized recycle delays of 1–5 seconds, spinal‐64 heteronuclear decoupling (at *ν*_rf_=85 kHz) and are a sum of 64–512 co‐added transients. Relative frequencies are reported with respect to 1 m NaCl used as an external secondary reference.

^23^Na and ^23^Na{^1^H} MQMAS experiments were acquired using a *z*filtered experiment with Fast Amplitude Modulated pulse train (2 loops) for the conversion step.^51^ The hard pulses were 4.5 and 2.2 μs (*ν*_rf_=83 kHz) and the selective pulse 9 μs (*ν*_rf_=14 kHz). Spectra were rotor‐synchronised in the indirect dimension and acquired at spinning rates of 5–10 kHz, with recycle delays of 1–5 seconds and spinal‐64 heteronuclear decoupling (at *ν*_rf_=85 kHz). For crystalline samples sufficient increments were collected to cover 8–10 ms of evolution in *t*_1_, whereas for the solid precipitate samples the signal decayed within 2 ms. All displayed MQMAS spectra were processed including a shearing transformation and the indirect dimension scaled following the C_3b_ convention.^52^

^27^Al MAS experiments were acquired using a Bloch‐decay sequence employing a 0.9 μs pulse (at *ν*_rf_=42 kHz), spinning rates of 10–14 kHz, optimized recycle delays of 2 s and are a sum of 32–128 co‐added transients. Relative frequencies are reported with respect to 1 m aluminium nitrate used as an external secondary reference.

^7^Li MAS experiments were acquired using a Bloch‐decay sequence employing a 0.8 μs pulse (at *ν*_rf_=50 kHz), spinning rates of 5–14 kHz, optimized recycle delays of 5 s and are a sum of 128 co‐added transients. Relative frequencies are reported with respect to 1 m LiCl, used as an external secondary reference.

### Scanning electron microscopy

Solid samples were pelletised with a 15–25 tonne manual hydraulic press (Specac) prior to analysis. The dried samples were mounted with double‐sided carbon adhesive tape onto 12.5 mm diameter aluminium stubs to be examined by scanning electron microscopy (SEM) and energy dispersive X‐ray analysis (EDX) at the York JEOL Nanocentre. To minimize charging for SEM imaging the samples were sputter coated for 5 min with platinum and palladium (15 nm) using a JEOL JFE‐2300HR high‐resolution fine coater (JEOL, USA). An extreme‐resolution analytical field emission SEM (JEOL JSM‐7800F, USA), operating at an acceleration voltage of 5 kV, was used for best resolution.

### Powder X‐ray diffraction

Powder XRD was performed using a Bruker D8 powder diffractometer equipped with a Cu source. A PSD Lynxeye detector in a Bragg–Brentano *θ*‐2 *θ* geometry was used and spectra were analysed using EVA software from Bruker. Samples were ground to a fine powder and analysed over 2 *θ*=5–90°, with a 0.0066° step size each averaged over 0.1 s per point for a total acquisition time of 23 min. Samples were analysed at room temperature. Generator voltage and current were set to 40 kV and 40 mA respectively. Reference data was obtained from the ICSD online database.^53^

### Inductively coupled plasma mass spectrometry (ICP‐MS)

Identification of sodium and aluminium was performed by ICP‐MS. Samples were digested in 5 mL of nitric acid (TraceSELECT solvent grade, Sigma–Aldrich) and then heated to 110 °C for three hours. After leaving to cool, the sample was dissolved in 100 mL of ultra‐pure water and diluted further if required. The samples were analysed with an Agilent 7700× ICP‐MS spectrometer, using nickel sample and skimmer cones. The analysis was run under helium. For sampling, the sample was taken up for 60 s, stabilised for 40 s, and washed for 60 s (with 5 % HCl for 30 s and 2 % HNO_3_ for 30 s). Each sample was run three times and the mean value of sodium and aluminium in ppm or ppb was obtained.

### Determination of carbonate and hydrogen carbonate concentrations in the electrolyte^31^

A phenolphthalein solution was made by dissolving phenolphthalein (1.25 g) in ethanol (125 mL) and water (125 mL). A methyl orange/indigo carmine solution was prepared by dissolving methyl orange (0.25 g) and indigo carmine (0.625 g) in water (250 mL). A 0.1 m HCl solution was prepared from a 12 m HCl solution. The 0.1 m HCl was standardised with 0.1 m sodium carbonate prior to dilution. Thus, pre‐heated sodium carbonate (5.3 g) was dissolved in water (500 mL) and then titrated with 0.1 m HCl with the methyl orange/indigo carmine solution as indicator to determine the HCl concentration. A 0.01 m NaOH solution (carbonate free) was prepared by adding NaOH (25 g) to water (25 mL) and the solution left to settle. An aliquot (0.325 mL) of this solution was then added to water (500 mL). This solution was standardised in 10 mL aliquots using 0.01 m HCl with phenolphthalein solution as indicator.

To determine the concentration of hydrogen carbonate in the electrolyte, a measured excess of 0.01 m NaOH was added to a known volume of electrolyte, along with a few drops of phenolphthalein, to convert all hydrogen carbonate anions into carbonate anions following the literature procedure. An excess of 10 % BaCl_2_ solution was then added to form BaCO_3_, whilst the analyte was heated to 70 °C for 1 min. The solution was then taken off the heat and titrated immediately with 0.01 m HCl, until the solution turned from pink to colourless. The added volume of 0.01 m HCl was used to determine the excess NaOH added and to calculate the exact volume of NaOH required to convert all hydrogen carbonate anions into carbonate anions. Hence, the moles of carbon dioxide in the electrolyte could be calculated.

To determine the concentration of carbonate in the electrolyte, a few drops of methyl orange/indigo carmine indicator were added to a measured aliquot of fresh electrolyte to turn the solution grey. The solution was then titrated with 0.01 m HCl to turn any carbonate or hydrogen carbonate anions into carbonic acid until the solution turned violet. The added volume of 0.01 m HCl was used to calculate the combined concentration of carbonate and hydrogen carbonate and the carbonate concentration could be obtained by subtracting the already determined hydrogen carbonate concentration.

### Seawater collection and analysis

Seawater (3 L) was collected from the coast of Whitby, North Yorkshire, UK, at 54° 29′ 31.0“ N 0° 36′ 28.8” W (Figure S30). The water was collected from the East beach during early July 2015. All 3 L were filtered through cellulose nitrate membrane filters (GC Whatman, pore size 0.2 μm, 47 mm diameter) to remove any sand or solid particulates. The seawater was kept in 100 mL falcon sealable bottles at −15 °C, to prevent the growth of any bacterial or fungi. The seawater was left to warm up to room temperature before any electrochemical run. Seawater was analysed with ICP‐MS for the presence of other trace elements (Table S5).

## Conflict of interest

*The authors declare no conflict of interest*.

## Supporting information

As a service to our authors and readers, this journal provides supporting information supplied by the authors. Such materials are peer reviewed and may be re‐organized for online delivery, but are not copy‐edited or typeset. Technical support issues arising from supporting information (other than missing files) should be addressed to the authors.

Supplementary
