# Supplementary material for: Capacitance‐Assisted Sustainable Electrochemical Carbon Dioxide Mineralisation
Source: ChemSusChem. 2017 Dec 12;11(1):137–48. doi: 10.1002/cssc.201702087 (PMC5814831; doi:10.1002/cssc.201702087)
Supplement: Supplementary file 1 — Supplementary [file CSSC-11-137-s001.pdf]

CHEMISTRY & SUSTAINABILITY
CHEMSUSCHEM
ENERGY & MATERIALS

Supporting Information

Capacitance-Assisted Sustainable Electrochemical Carbon Dioxide Mineralisation

Katie J. Lamb+,[a] Mark R. Dowsett+,[a] Konstantinos Chatzipanagis,[b] Zhan Wei Scullion,[b] Roland Kröger,[b] James D. Lee,[a] Pedro M. Aguiar,*[a] Michael North,*[a] and Alison Parkin*[a]

cssc_201702087_sm_miscellaneous_information.pdf

Capacitance Assisted Sustainable Electrochemical Carbon Dioxide Mineralisation

Katie J. Lamb,†[a] Mark R. Dowsett,†[a] Konstantinos Chatzipanagis,¥[b] Zhan Wei Scullion,[b] Roland Kröger,[b] James D. Lee,[a] Pedro M. Aguiar,§*[a] Michael North*[a] and Alison Parkin*[a]

a. Department of Chemistry, University of York, York, YO10 5DD, UK
b. Department of Physics, University of York, York, YO10 5DD, UK

*E-mail: pedro.aguiar@umontreal.ca, michael.north@york.ac.uk, alison.parkin@york.ac.uk

† These authors contributed equally
¥ Current address: GFZ-Helmholtz Zentrum (Interface Geochemistry group), Potsdam, Germany
§ Current address: Department of Chemistry, Université de Montréal, Montreal QC H3C 3J7, Canada

Original data on which this research is based is available at:
DOI: 10.15124/b0769e3b-c1e0-40e0-bdbd-b5d9c059cb02

Electrochemical cell photographs and technical diagram 2
Solid state analysis data 3
Control electrochemical experiments 12
Aluminium Pourbaix diagram 14
Varying the electrochemical cell components 15
Technical specification of the graphite used 18
Electrode calibration 18
Seawater collection and analysis 20
References 20

Electrochemical cell photographs and technical diagram

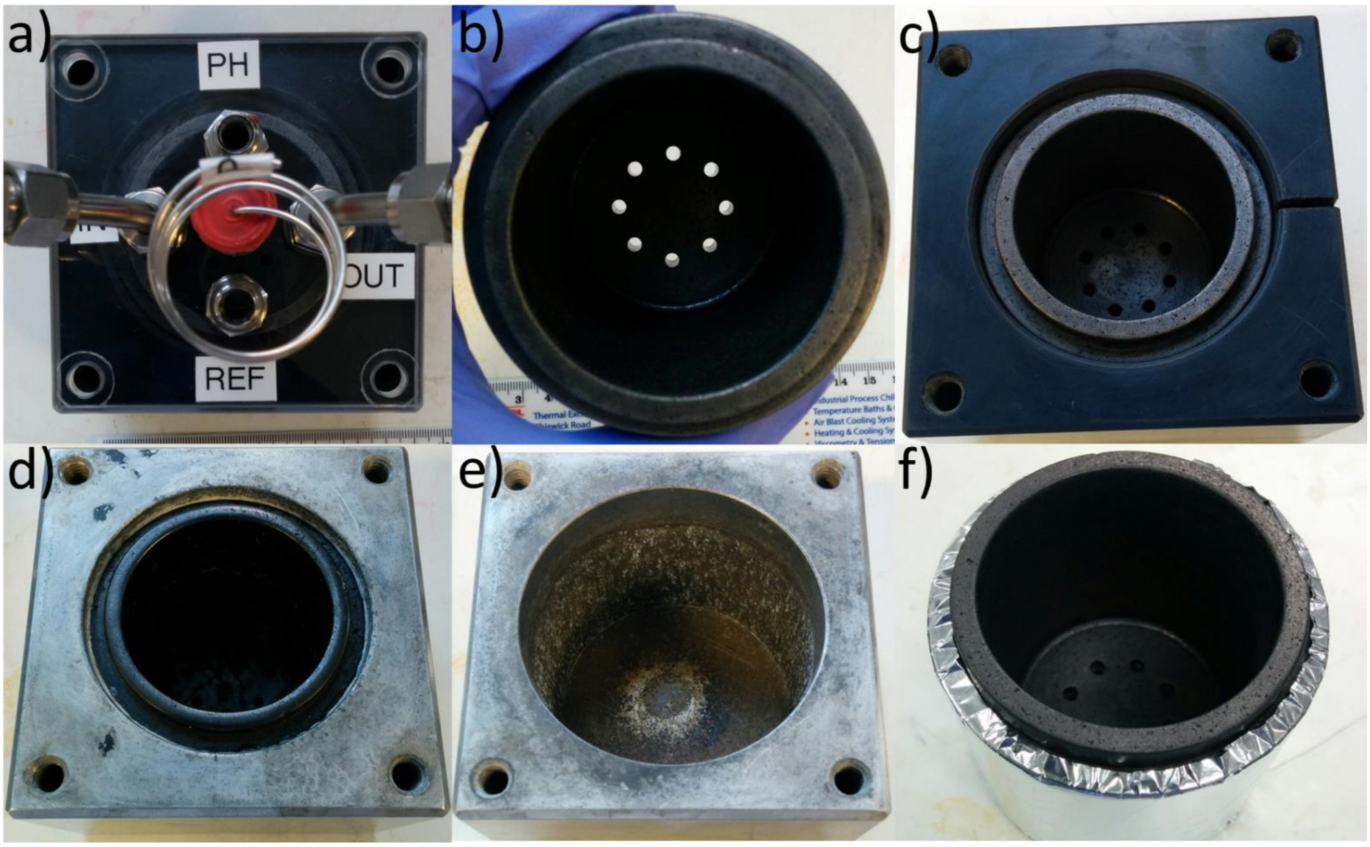

g)

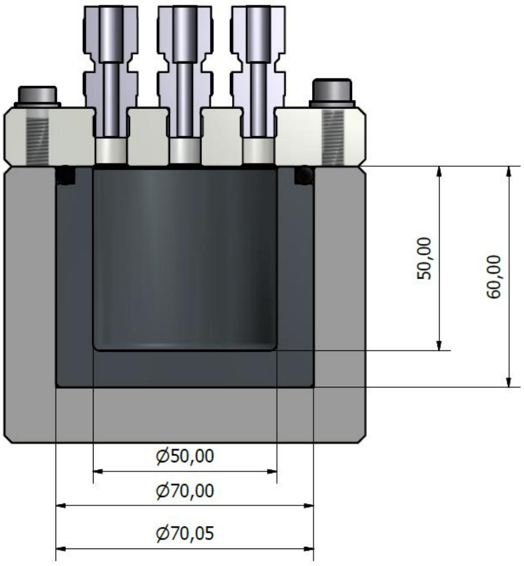

Figure S1: Electrochemical cells used for CO_2 capture featuring: a) the cell lid with ports for the cathode (center), a reference and pH probe and a gas inlet and outlet; b) a removable carbon cup with 8 x 3.2 mm holes drilled through the base, abbreviated simply as C; c) carbon cup in (b) held in an acrylic container; d) a carbon cup analogous to (b) irreversibly held inside a solid aluminium block, abbreviated as C+Al_{block} when used as a dual anode standard cell; e) an aluminium block without a carbon liner, abbreviated as Al_{block}; f) a removable carbon cup, (b), wrapped in aluminium foil for use in the acrylic cell in (c), abbreviated as C+Al_{waste}. g) technical diagram showing the dimensions of the carbon-aluminium cell.

Solid state analysis data

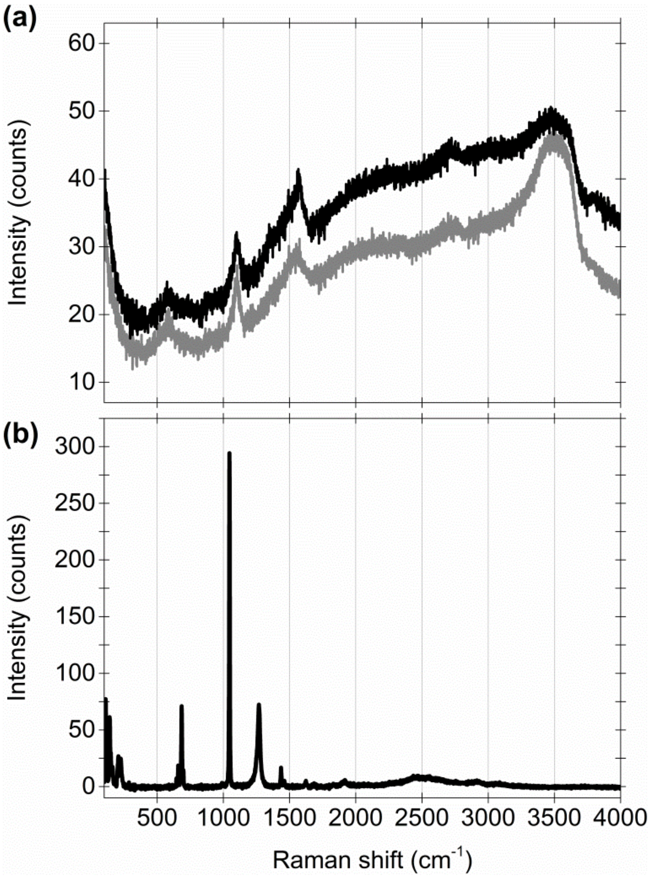

Figure S2: Raman analysis of (a) two different regions of isolated precipitate post-CO_2 capture in the graphite-aluminium anode standard cell and (b) identical measurement performed on a $NaHCO_3$ sample for comparison.

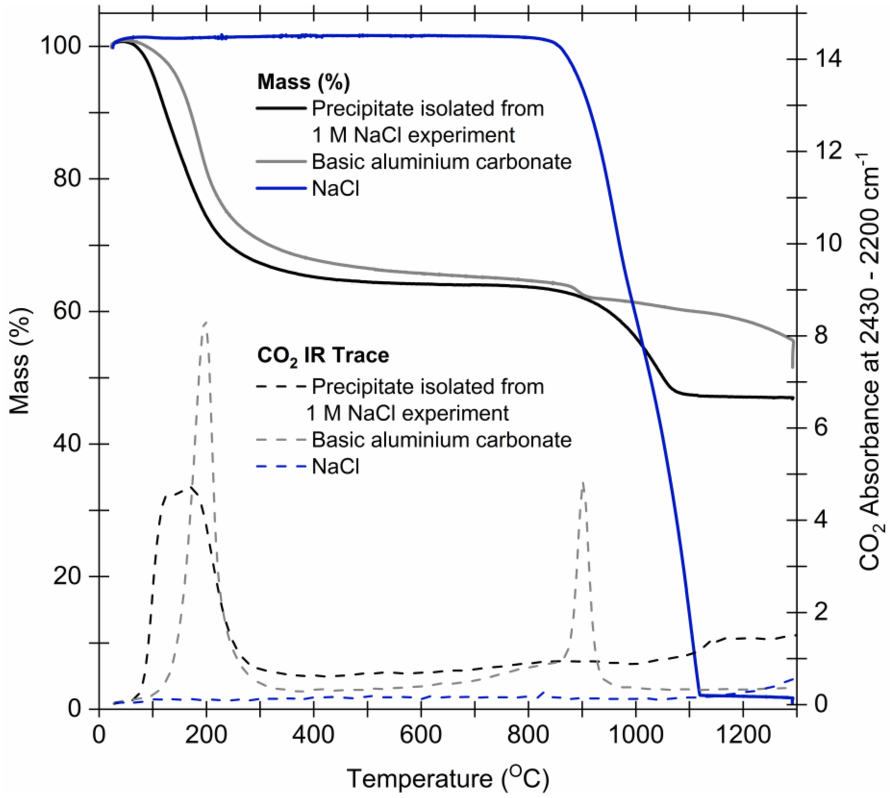

Figure S3: Comparing TGA-IR analysis of precipitate isolated post-CO_2 capture in the aluminium-graphite anode standard cell, commercial basic aluminium carbonate [$Al(CO_3)(HCO_3)$] and commercial sodium chloride. All measurements taken at a ramp rate of 10 °C min^{-1}.

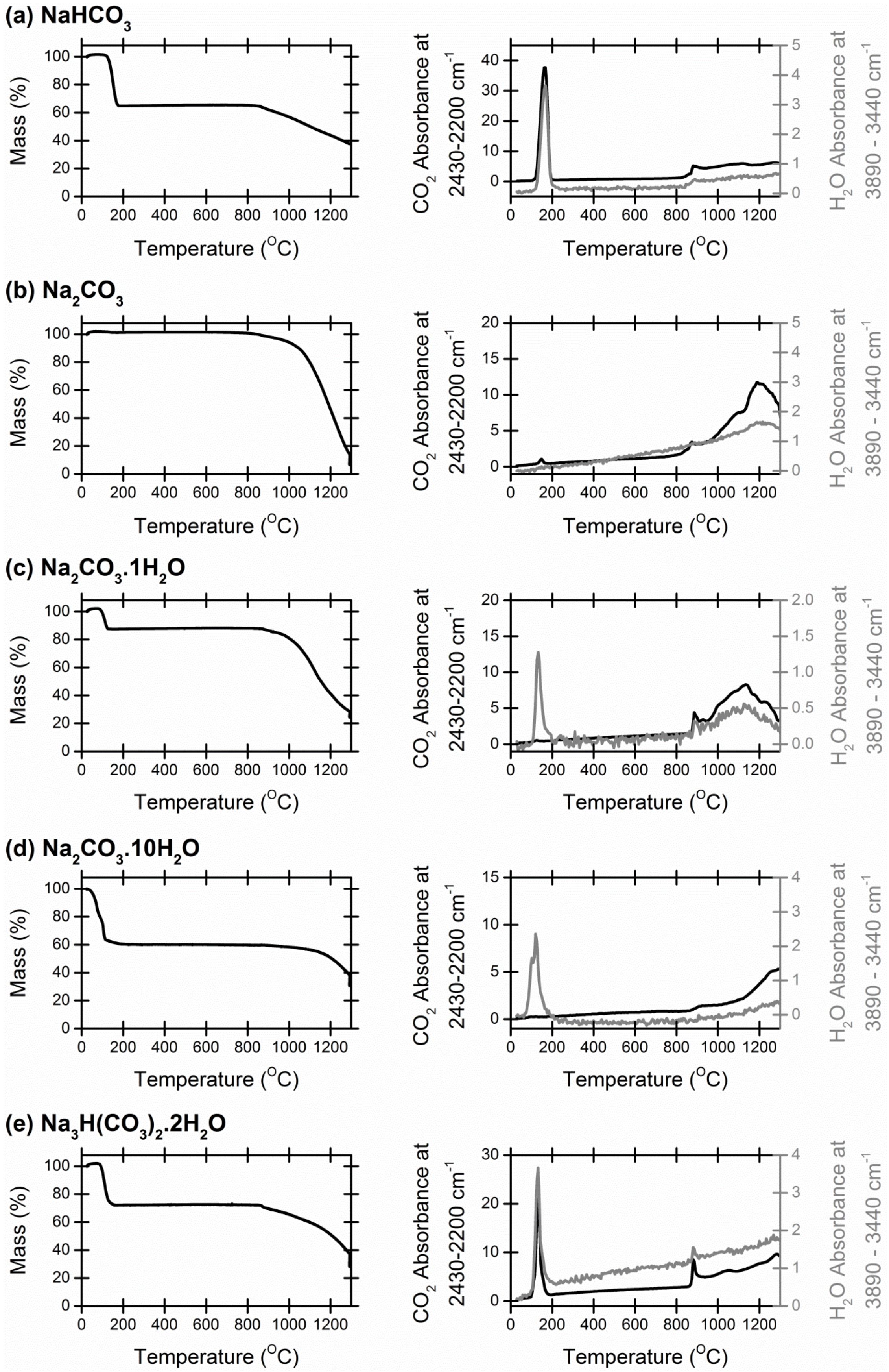

Figure S4: TGA-IR analysis of commercial sodium carbonate and hydrogencarbonate standards showing that CO_2 loss from hydrogen carbonates coccurs below 200 ^{o}C whilst CO_2 loss from carbonates occurs above 800 ^{o}C.

Table S1: Calibration data for quantitative TGA-IR relative to a $CaCO_3$ standard.

$CaCO_3$ mass / mg	CO_2 mass loss / mg	CO_2 absorbance-time peak area	Mass loss : peak area ratio
30.2	13.4	201.2	0.064
50.9	21.7	342.2	0.074
99.9	43.6	586.2	0.066
		Average	0.068 ± 0.006

(a)

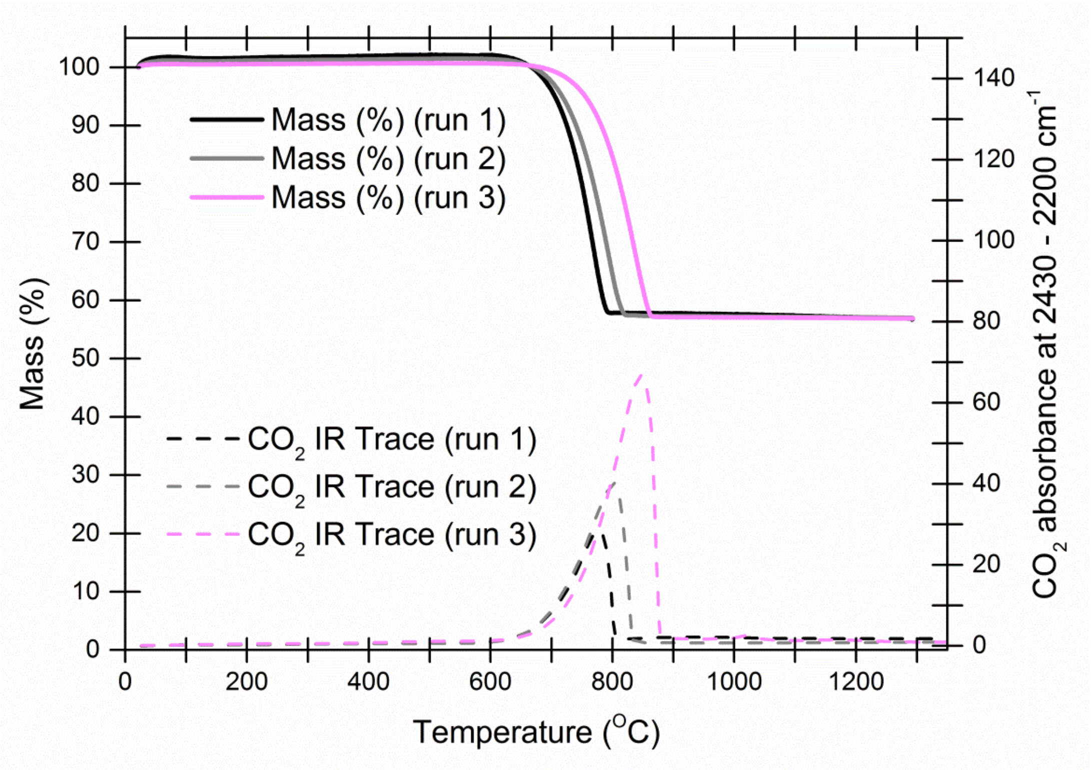

(b)

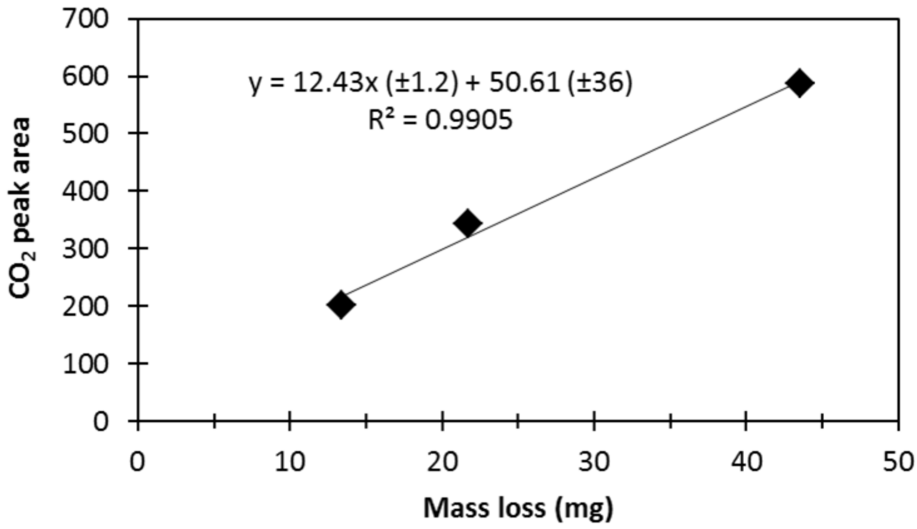

Figure S5: (a) TGA mass loss trace and corresponding CO_2 IR trace obtained for standard $CaCO_3$. (b) Resultant calibration graph relating IR CO_2 peak area (from 2430-2200 cm^{-1}) to mass loss for $CaCO_3$.

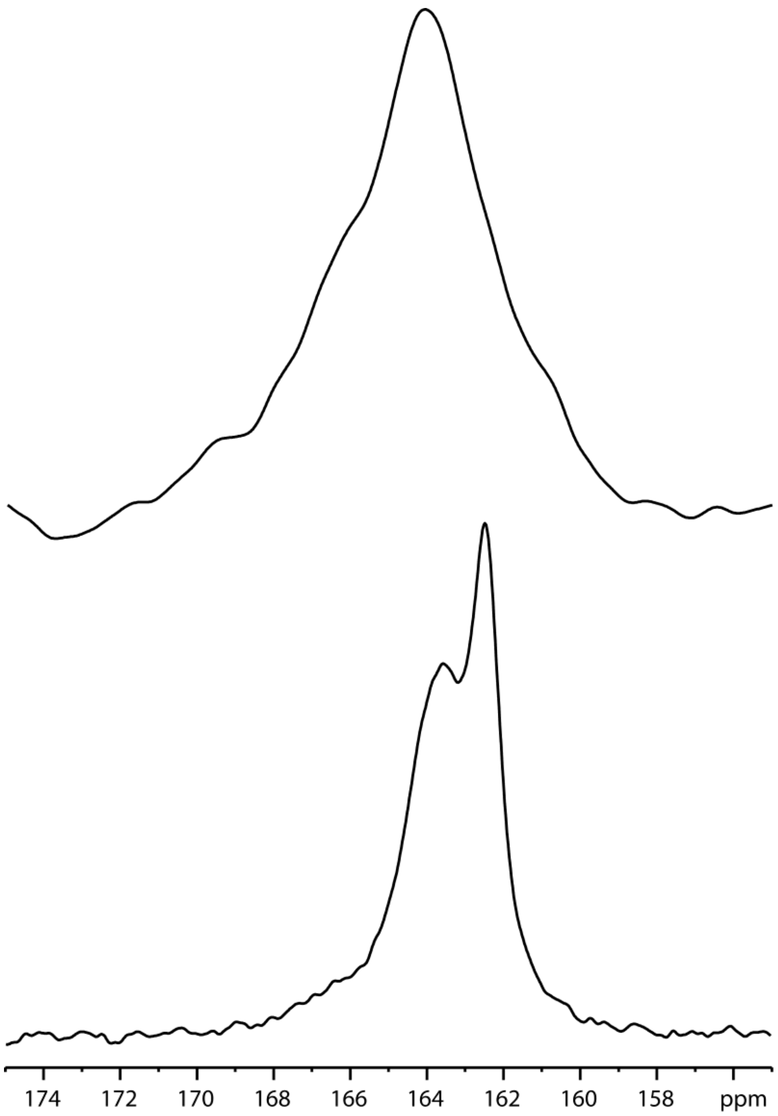

Figure S6: Top $^{13}C\{^1H\}$[1] CPMAS spectrum for the precipitate generated in 24-hour, 10 mA electrochemical carbon capture experiments using 1 M NaCl as electrolyte and the aluminium-graphite dual anode standard cell under 5% CO_2 / 95% N_2. The observed isotropic shifts of 162-164 ppm are typical of solids acquired from various electrochemical runs. Bottom $^{13}C\{^1H\}$ CPMAS spectrum of commercial $Al(CO_3)(HCO_3)$.

Table S2: ^{13}C MAS NMR isotropic shifts observed.

Sample	δ_{iso} (ppm)
$NaHCO_3$	164.8
Na_2CO_3	
signal 1	170.9
signal 2	170.7
$Na_2CO_3{\cdot}1H_2O$	172.3
$Na_2CO_3{\cdot}10H_2O$	169.0
$Na_3(HCO_3)(CO_3){\cdot}2H_2O$	167.8
$Al(CO_3)(HCO_3)$	
signal 1	164.3
signal 2	162.4
CO_2 capture solid	163

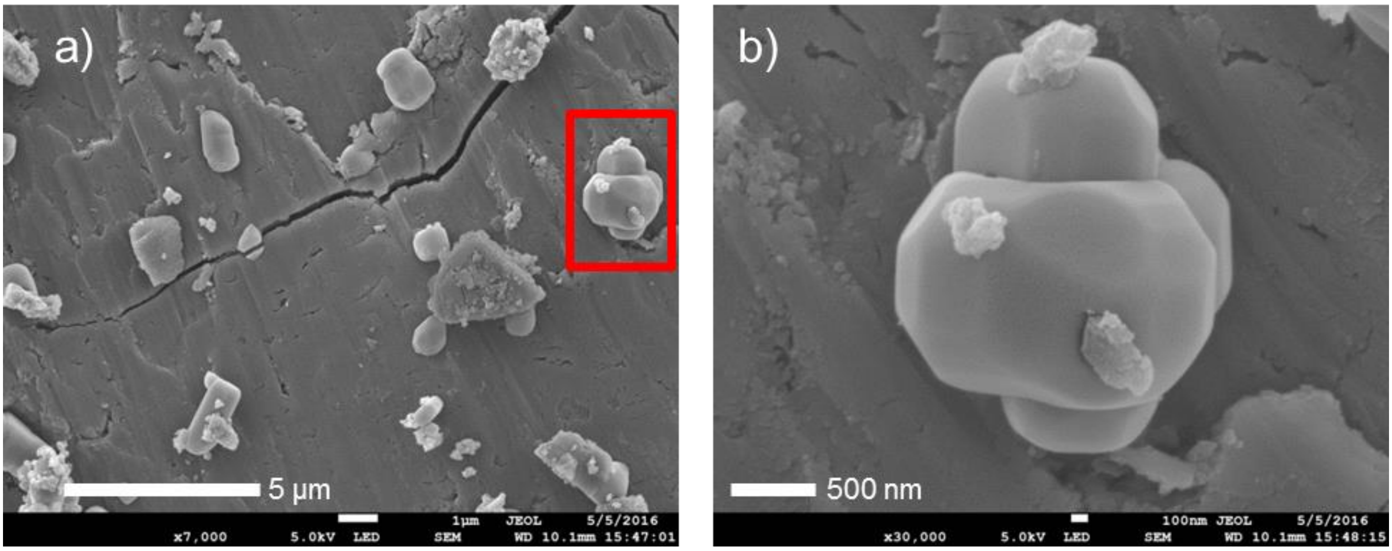

Figure S7. SEM analysis of precipitate isolated following CO_2 capture in the aluminium-graphite anode standard cell. (a) Shows small crystallites on the surface of the bulk material, (b) a closer view of the crystallite highlighted in the red square of panel a.

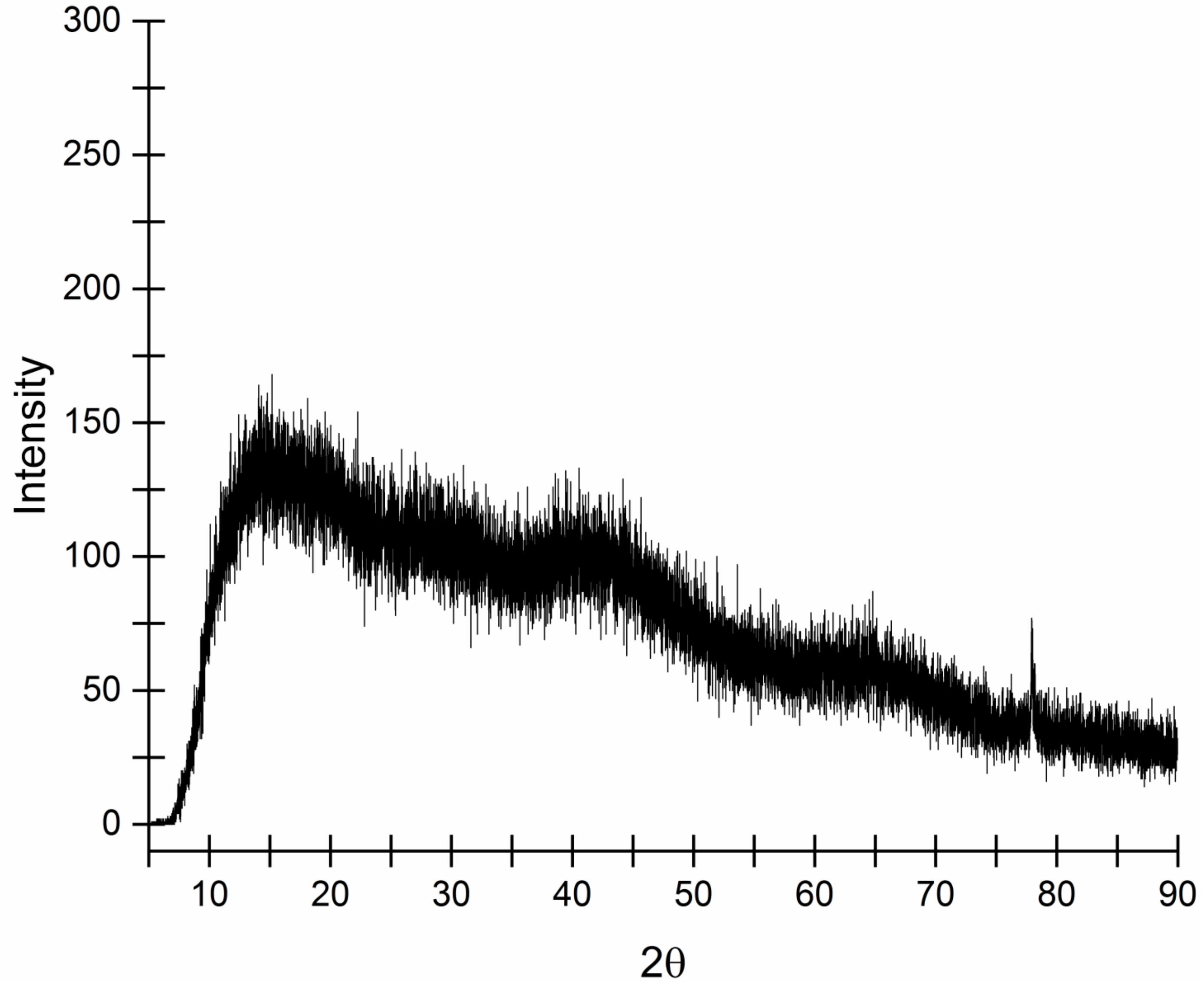

Figure S8. Powder X-ray diffraction data acquired after extensive washing of the solid precipitate with cold water to remove all sodium chloride.

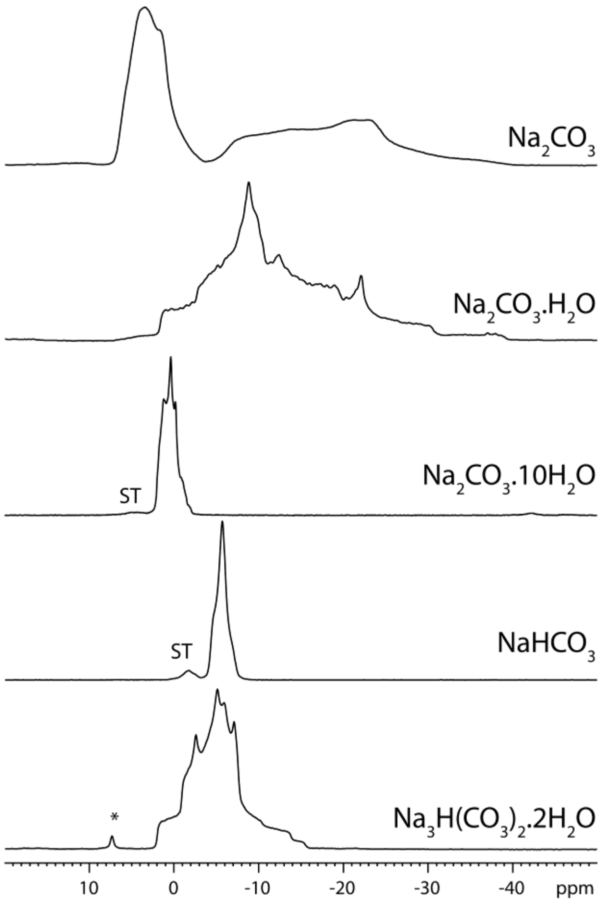

Figure S9: ^{23}Na MAS-NMR spectra of a series of sodium carbonate standards. Signals arising from the satellite transitions (ST) are marked where visible. A small amount of NaCl in the sesquicarbonate is marked by an asterisk. ^{23}Na 3QMAS also revealed a small amount (ca. 10% based on fits of the MAS lineshape) of sodium hydrogen carbonate in this sample, which was also observed in the ^{13}C spectrum of this sample.

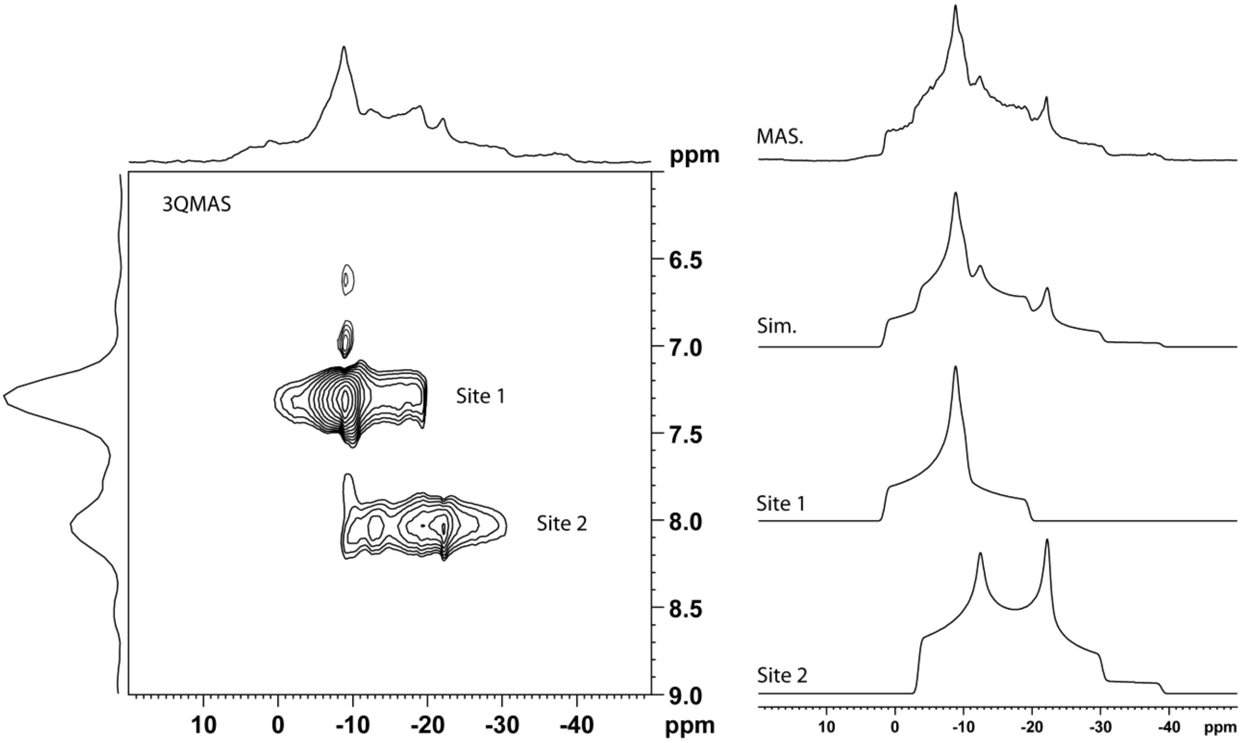

Figure S10: ^{23}Na 3QMAS spectrum (left) of $Na_2CO_3.H_2O$ showing resolution of the two overlapping signals along with ^{23}Na MAS and simulation of the lineshape taking into account the two signals.

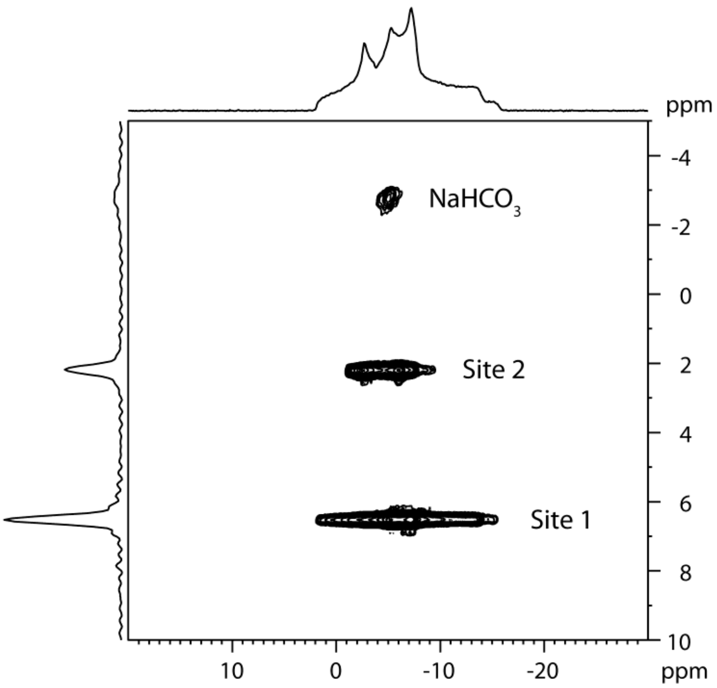

Figure S11: ^{23}Na 3QMAS spectrum of $Na_3H(CO_3)_2.2H_2O$ showing resolution of the two crystallographically distinct sites[2] along with sodium hydrogen carbonate impurity.

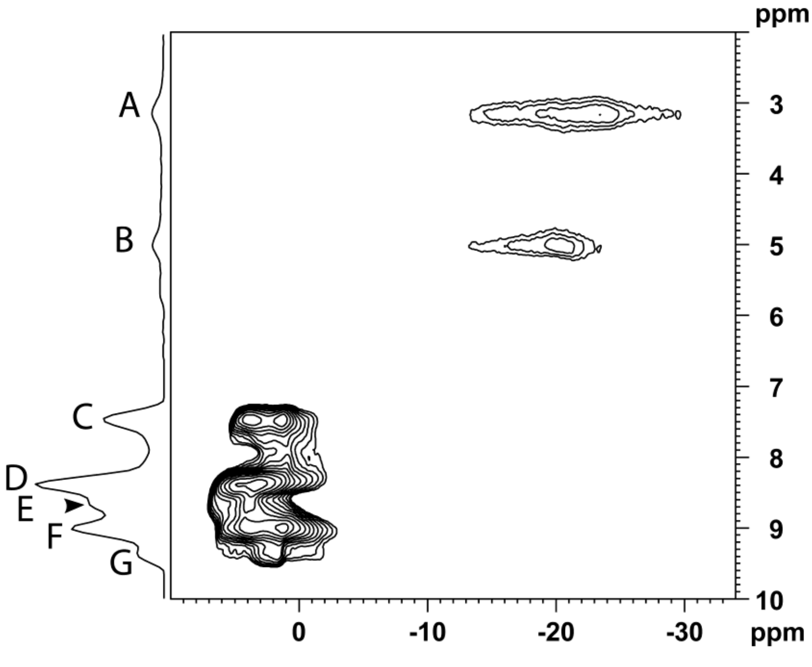

Figure S12. ^{23}Na 3QMAS spectrum of Na_2CO_3. A series of signals could be resolved to the large number of unique sites present in the incommensurately modulated structure of sodium carbonate.[3]

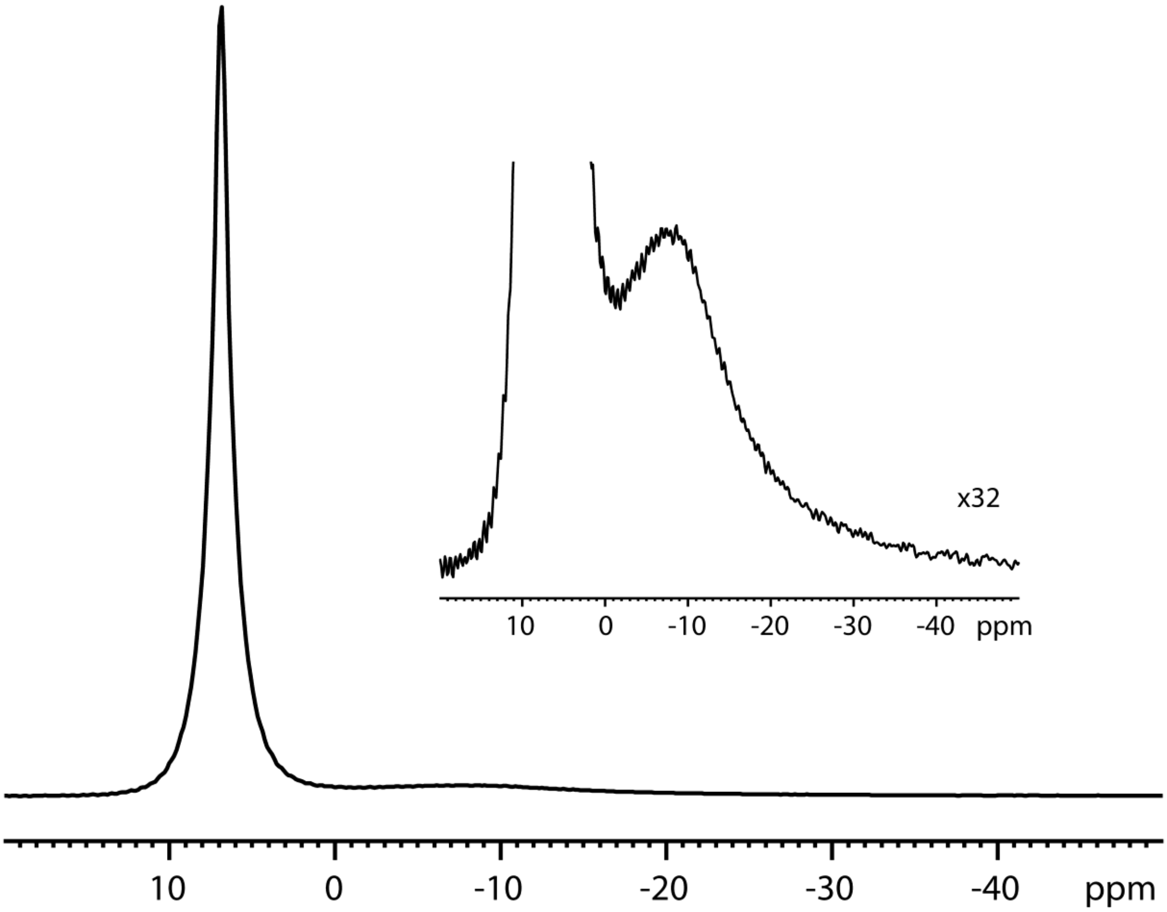

Figure S13. ^{23}Na MAS spectra of the solid precipitates generated in 24-hour, 10 mA electrochemical carbon capture experiments using 1 M NaCl as electrolyte and the aluminium-graphite dual anode standard cell. The amount of NaCl (δ = 7 ppm) co-precipitating varied from sample to sample. An inset of the upper spectrum (x32) shows the carbonate component more clearly.

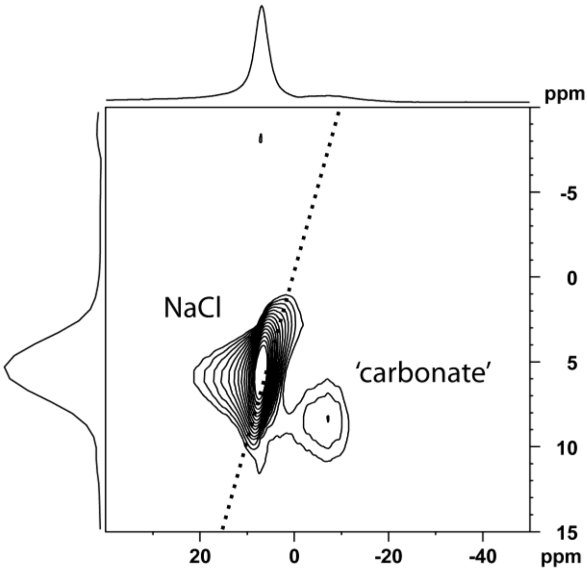

Figure S14. ^{23}Na 3QMAS spectra of solid precipitates with differing amounts of NaCl. The slope of the dashed line corresponds to the chemical shift contribution to the position in the 'isotropic' (vertical) dimension. Elongation along this slope indicates a distribution of isotropic chemical shifts.[4]

Table S3. ^{23}Na NMR parameters for sodium carbonate standards.

Sample	δ_{iso} (ppm)	C_Q (MHz)	η	P_Q (MHz)
$NaHCO_3$	-4.3	0.68	0.95	0.88
$Na_2CO_3 \cdot 1H_2O$[a]				
site 1	1.5	1.80	1.00	2.40
site 2	-1.8	2.54	0.44	2.70
$Na_2CO_3 \cdot 10H_2O$[a]				
site 1	2.2	0.77	0.48	0.80
site 2	1.9	0.81	0.65	0.87
$Na_3(HCO_3)(CO_3) \cdot 2H_2O$[a]				
site 1	2.0	1.70	0.75	1.90
site 2	-0.2	1.33	0.26	1.39
Na_2CO_3[a,b]				
signal A	-6.2	-	-	2.73
signal B	-4.5	-	-	2.73
signals C-G	5.6-6.9	-	-	1.20-1.40
CO_2 capture solid[a]	1.2	-	-	1.54

[a] Parameters extracted from [23]Na 3QMAS experiments.

[b] The structure of Na_2CO_3 is incommensurately modulated (aperiodic) resulting in signals representing the distribution of sodium environments. Thus even ‘resolved’ signals belong to a distribution of sites in the crystal. No attempt has been made herein to ascribe the observed signals to the sites within crystal.

Control electrochemical experiments

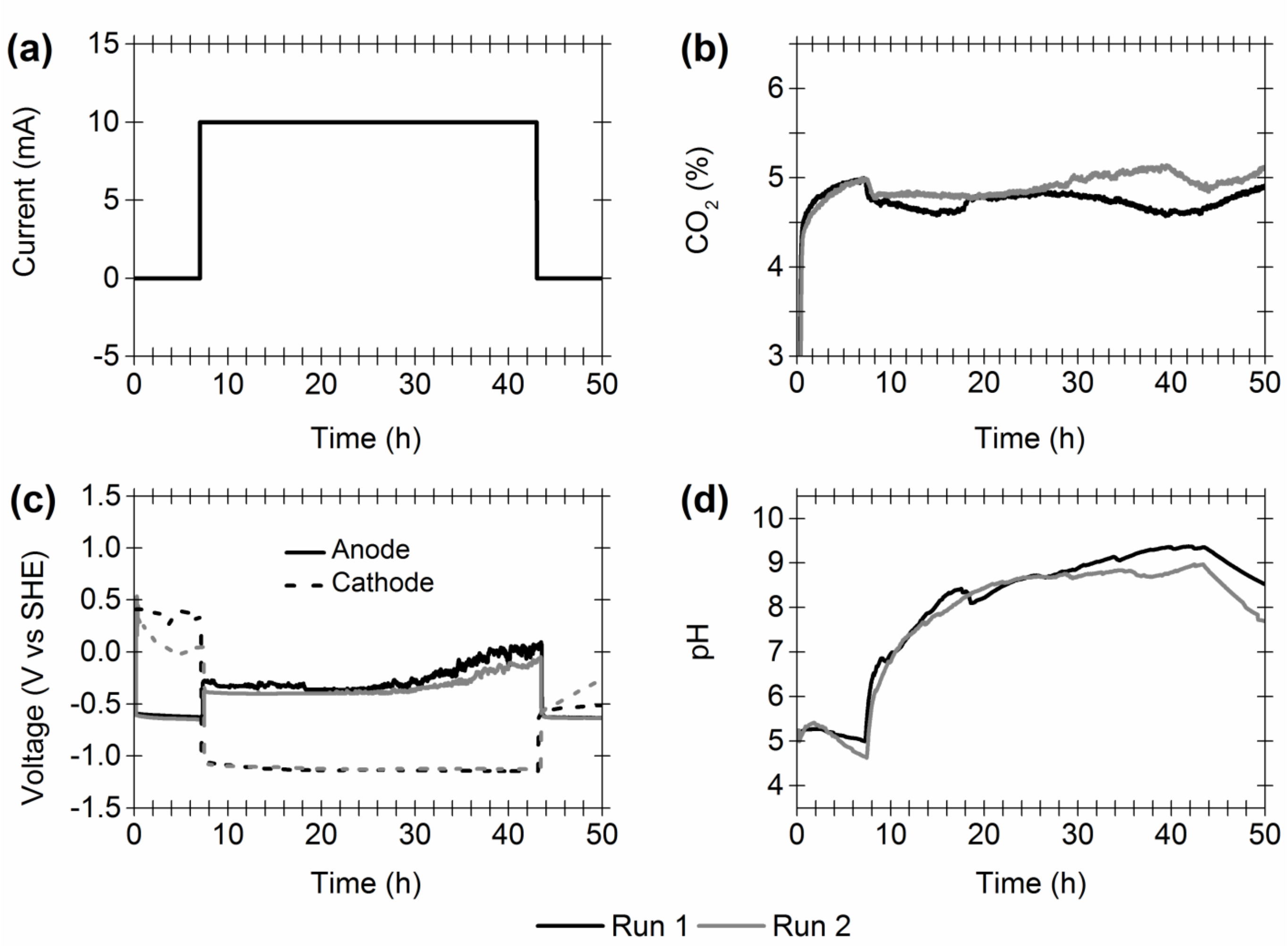

Figure S15. Carbon dioxide fixation using a 10 mA current over 36 h with 1 M aqueous NaCl as electrolyte in the dual-material aluminium-graphite anode cell, for two different experimental runs differentiated by line colour. (a) Current-time trace; (b) carbon dioxide content in the exit gas stream; (c) the voltage of the aluminium-graphite anode (solid line) and platinum cathode (dashed line); (d) solution pH.

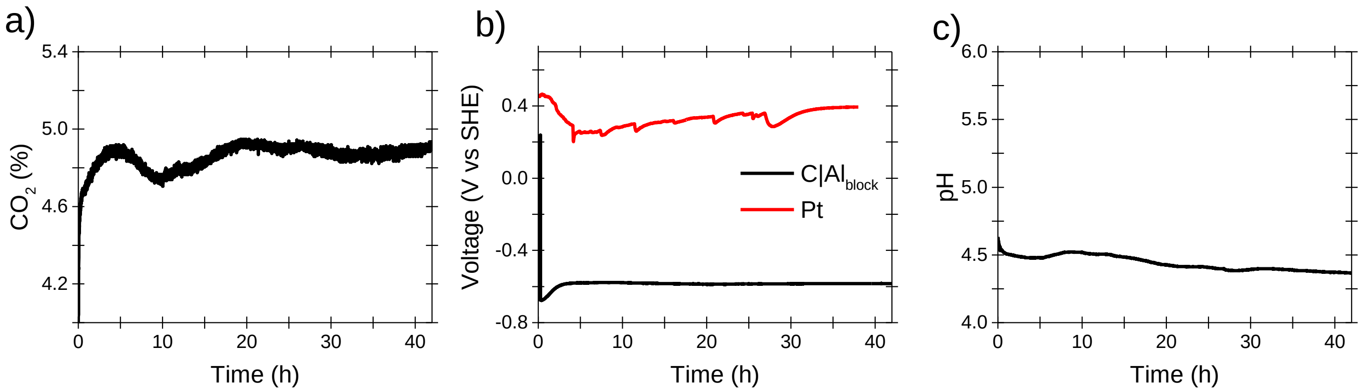

Figure S16. Standard cell at open circuit potential for 40 h at 25 °C with 1 M NaCl electrolyte under 5% CO_2 / 95% N_2. No current step applied to give instrument traces for: a) exit gas stream CO_2; b) electrode voltages; c) pH.

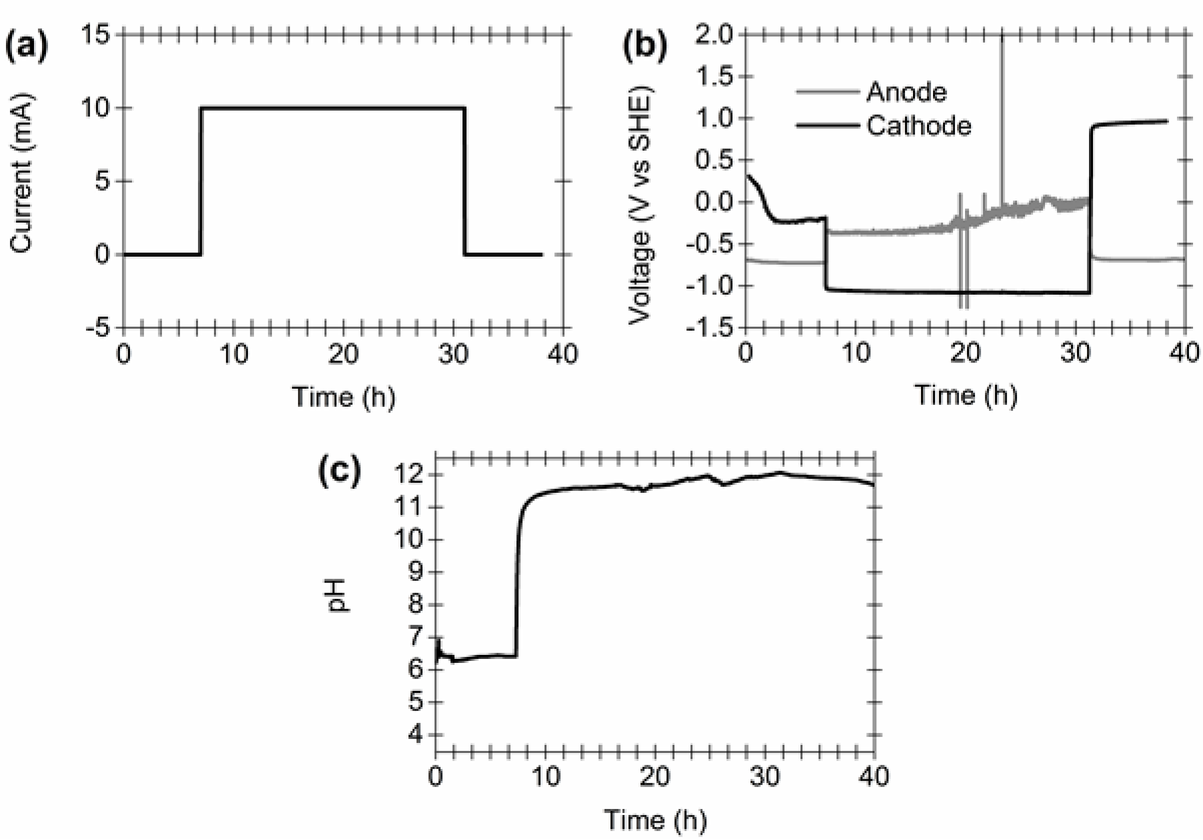

Figure S17. Standard cell with a 10 mA current step for 24 h with a pre- and post-current equilibration step at open circuit potential at 34 °C. 1 M NaCl electrolyte under a 100% N_2 atmosphere. Instrument traces shown for: a) applied current; b) voltage versus SHE, obtained using a calibrated Ag/AgCl reference; c) pH.

Aluminium Pourbaix diagram

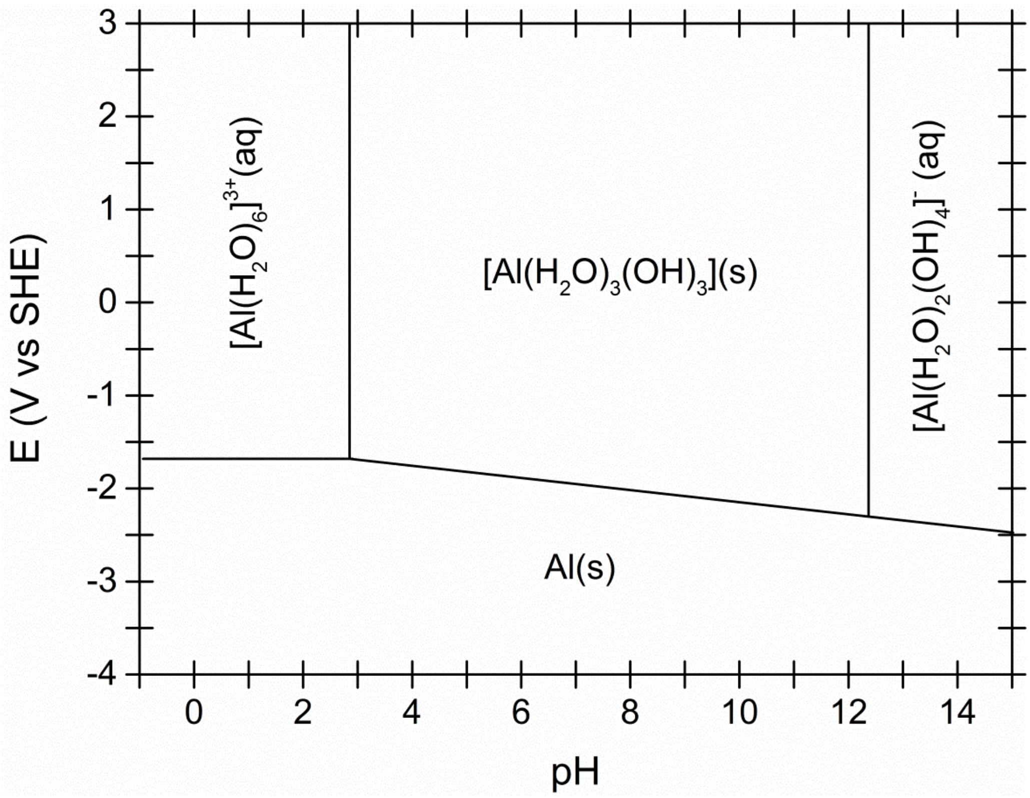

0

Figure S18. Pourbaix plot for aluminium at different voltages and pH levels. The small regions of $Al(H_2O)_5(OH)^{2+}$ and $Al(H_2O)_4(OH)_2^+$ have been omitted for simplicity. Reproduced as per Deltombe and Pourbaix.[5]

Varying the electrochemical cell components

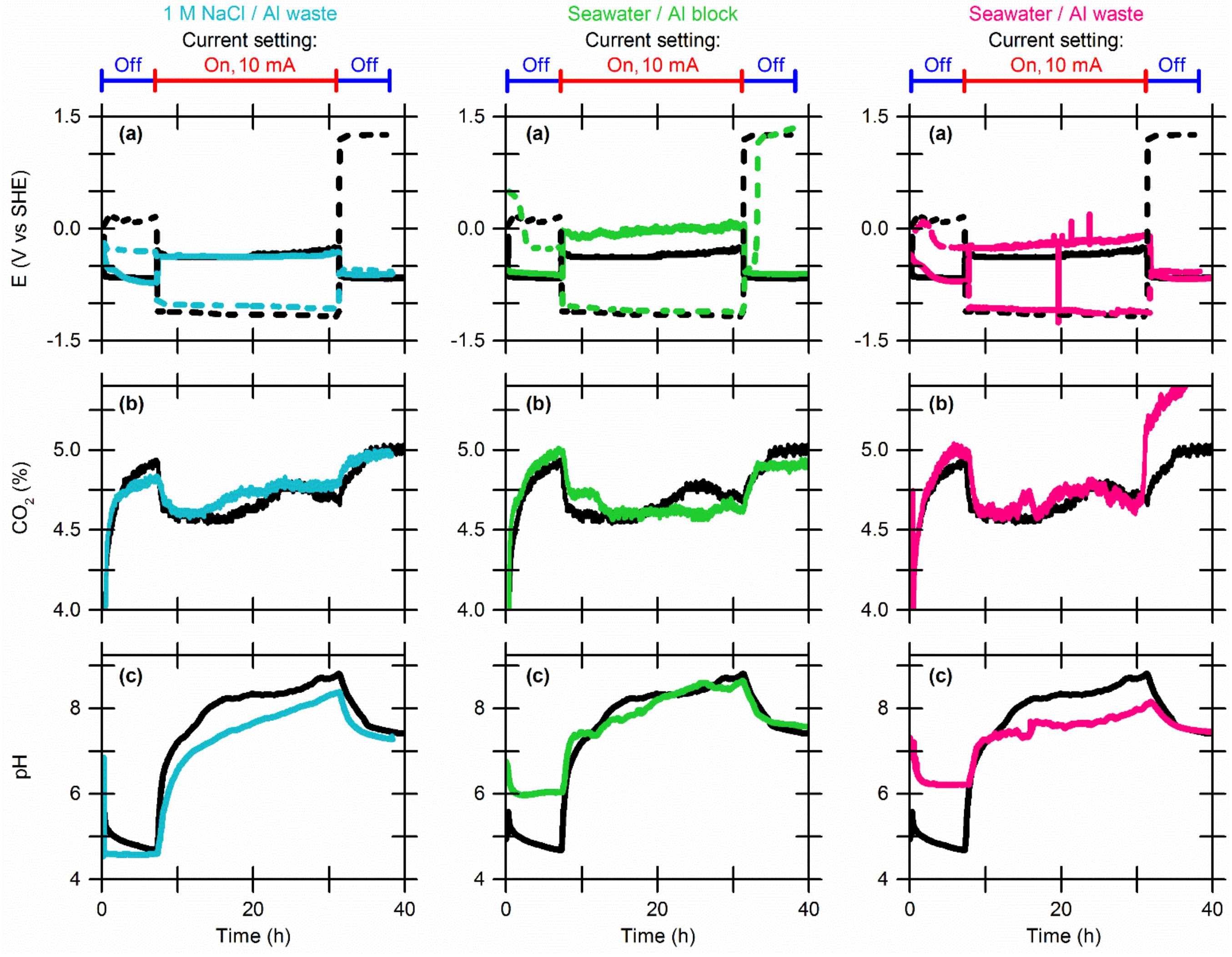

Figure S19. Comparing CO_2 capture with 1 M NaCl in the dual aluminium-graphite anode standard cell (black lines) to “waste” aluminium (aluminium foil, top left, blue lines), seawater (top middle, green lines) and both combined (top right, red lines). (a) Anode (solid line) and cathode (dashed line) voltages. (b) Carbon dioxide content in the exit gas stream. (c) Changes in solution pH. Experiments performed at 34 °C with a gas flow of 5% CO_2 / 95% N_2 at 14 mL min^{-1}.

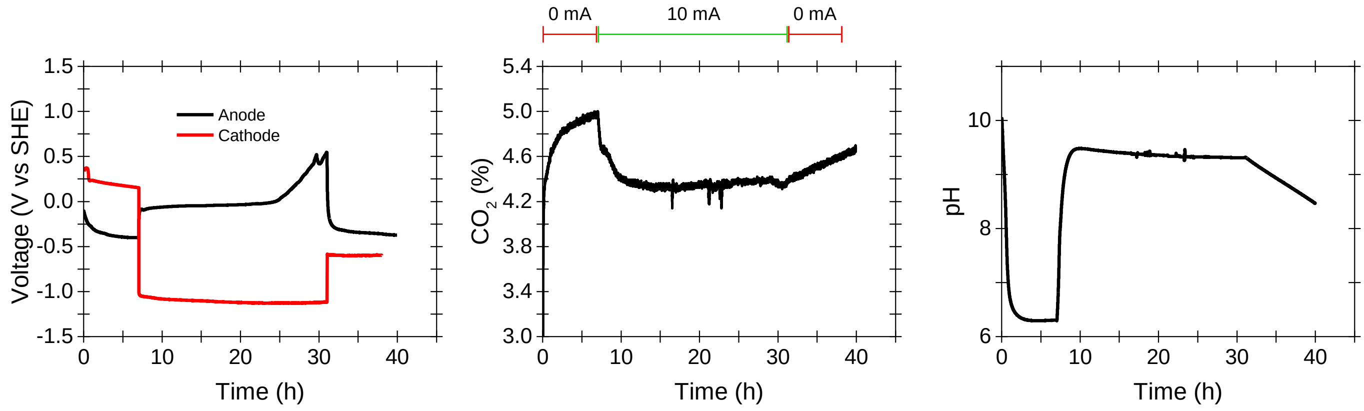

Figure S20. C+Fe$_{disk}$ dual material anode and Pt cathode cell with a 10 mA current step for 24 h with a pre- and post-current equilibration step at open circuit potential. 1 M NaCl electrolyte at 25 °C under a 5% CO_2 / 95% N_2 atmosphere. Instrument traces shown for: a) voltage versus SHE, obtained using a calibrated Ag/AgCl reference; b) exit gas stream CO_2; c) pH.

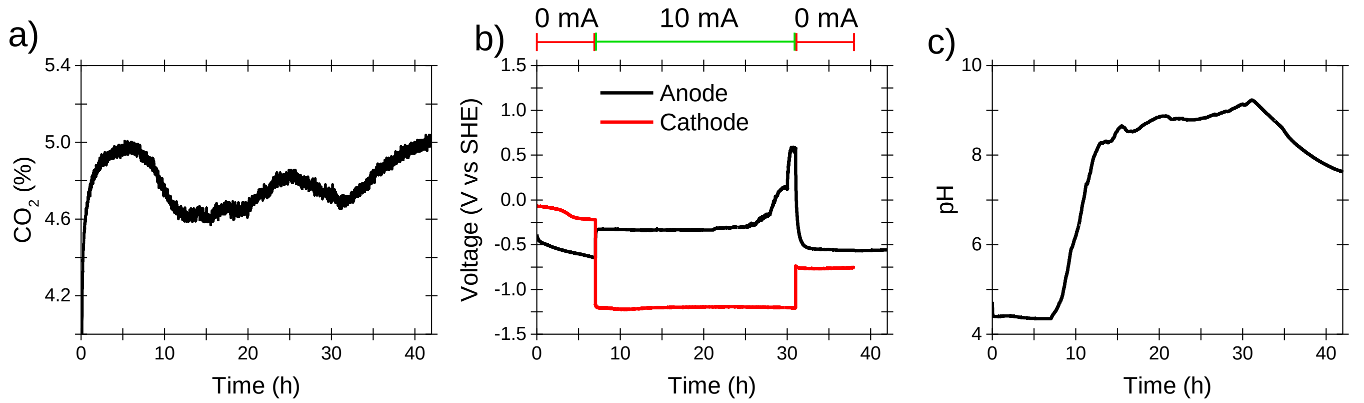

Figure S21. C+Al$_{waste}$ dual material anode and Ni cathode cell with a 10 mA current step for 24 h with a pre- and post-current equilibration step at open circuit potential. 1 M NaCl electrolyte at 25 °C under a 5% CO_2 / 95% N_2 atmosphere. Instrument traces shown for: a) exit gas stream CO_2; b) voltage versus SHE, obtained using a calibrated Ag/AgCl reference; c) pH.

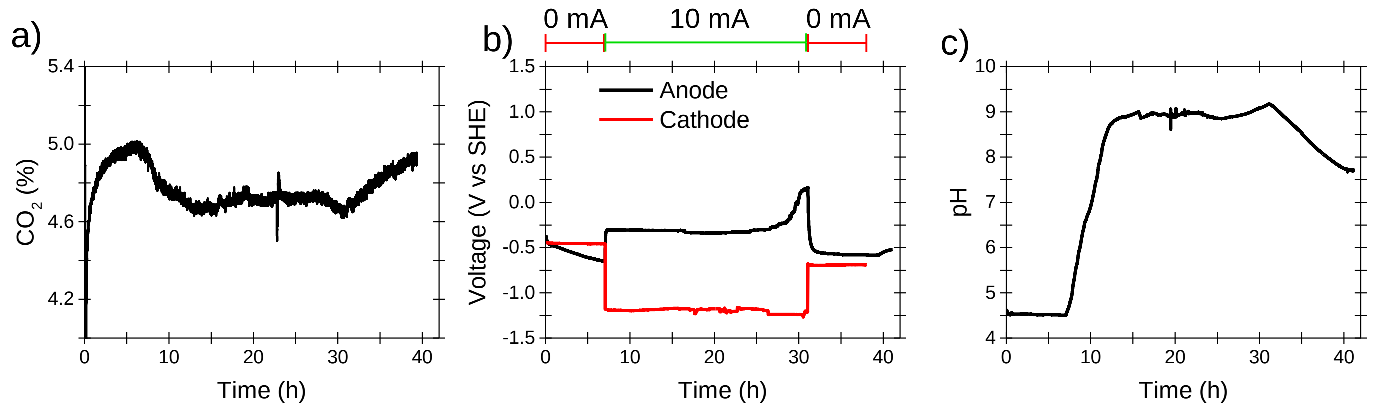

Figure S22. C+Al$_{waste}$ dual material anode and Fe cathode cell with a 10 mA current step for 24 h with a pre- and post-current equilibration step at open circuit potential. 1 M NaCl electrolyte at 25 °C under a 5% CO_2 / 95% N_2 atmosphere. Instrument traces shown for: a) exit gas stream CO_2; b) voltage versus SHE, obtained using a calibrated Ag/AgCl reference; c) pH.

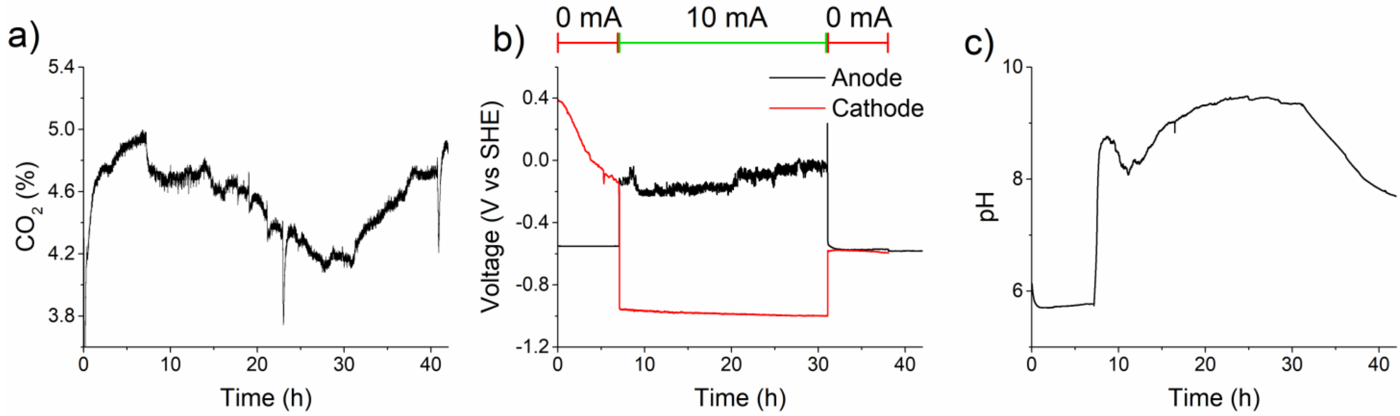

Figure S23. CO_2 capture with a 1 M NaCl electrolyte obtained by adding NaCl to sea water. (a) Carbon dioxide content in the exit gas stream. (b) Anode (black line) and cathode (red line) voltages. (c) Changes in solution pH. Experiments performed at 34 °C with a gas flow of 5% CO_2 / 95% N_2 at 14 mL min^{-1}.

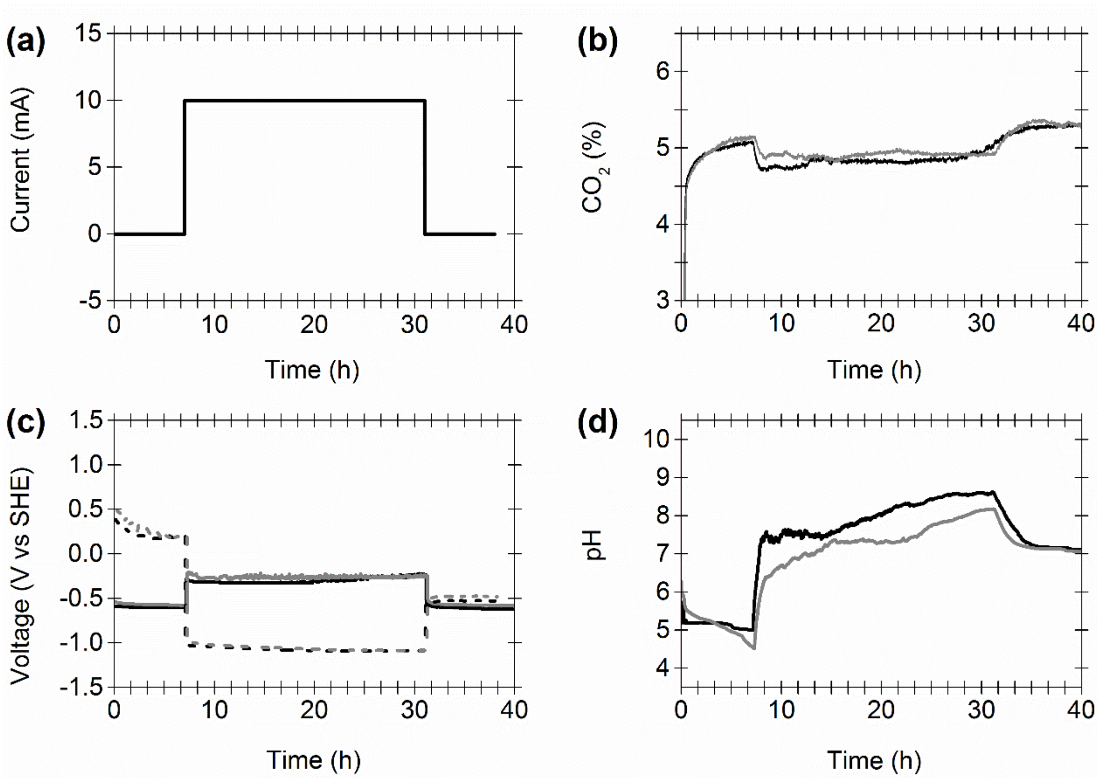

Figure S24. Comparing CO_2 capture in the aluminium-graphite anode standard cell using 1 M aqueous NaCl (black line) and 1 M aqueous LiCl (grey line). Instrument traces shown for: a) applied 10 mA current; b) CO_2 content in the exit gas stream; c) resultant changes to the voltage of the anode (solid line) and cathode (dashed line); d) Corresponding changes in solution pH. Cell at 34 °C under a continuous gas flow of 5% CO_2 / 95% N_2 at 14 mL min^{-1} CO_2.

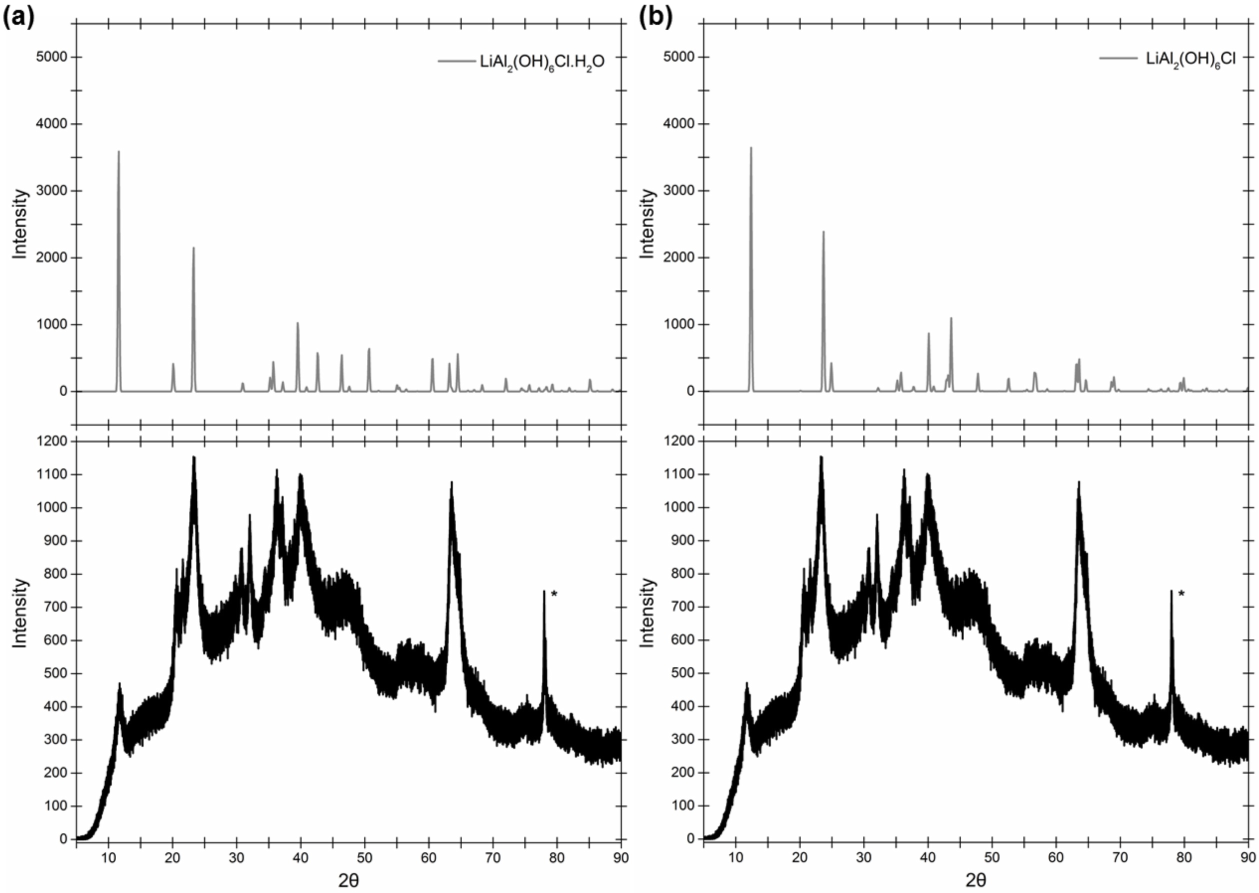

Figure S25: Comparing powder XRD spectra of the precipitate isolated post-CO_2 capture in the aluminium-graphite anode cell using 1 M LiCl (bottom (both), black line) with (a) $LiAl_2(OH)_6Cl.H_2O$ (grey line) and (b) $LiAl_2(OH)_6Cl$ (grey line) sample data.[6] * refers to the metal plate (Al) used to hold the sample during analysis.

Technical Data
Medium Grained Graphite

Extract from the Olmec programme of extruded grades

OLMEC ADVANCED MATERIALS

Grade	Bulk Density (g/cm3)	Porosity (%)	Spec. el. Resistance (Ω mm²/m)	Compressive Strength (N/mm2)	Bending Strength (N/mm2)	Ash Content (%)	Thermal Expansion Coefficient (10-6/K)	Thermal Conductivity (W/m K)	Max. Grain Size (mm)	Standard Sizes (mm)
MCCA	1.78 - 1.82	11 - 14	6 - 8	60	38 - 42	<0.1	2.0 - 2.5	120 - 140	0.35	∅ 3 .. 9
MCCA	1.76 - 1.80	12 - 15	6 - 8	35 - 40	20 - 25	<0.1	1.8 - 2.5	120 - 140	0.6	∅ 10 .. 25
MCCA	1.72 - 1.75	14 - 17	6 - 8	30 - 40	20 - 25	<0.1	1.8 - 2.5	120 - 140	0.6	∅ 30 .. 105
MCCA	1.70 - 1.73	16 - 19	6 - 8	26 - 32	17 - 20	<0.1	1.6 - 2.2	120 - 140	0.8	∅ 110 .. 700
MCCA	1.66 - 1.73	18 - 20	5 - 8	22 - 28	16 - 19	<0.1	1.6 - 2.2	120 - 140	0.8	Plates**
MRCM	1.76 - 1.80	12 - 15	4 - 5	50	25	<0.25	1.3 - 1.6	150 - 180	2	∅ 200 .. 300
VDN	1.67 - 1.73	17 - 20	5 - 7	23 -28	10 - 14	<0.8	1.5 - 2.0	130 - 150	10 - 20	∅ 300 .. 600

* In direction of extrusion
** Sizes in mm: 108 x 495 x 1100, 1800; 140 x 428 x 1100; 120 x 330 x 1100; 170 x 375 x 1100; 400 x 400 x 1800; 500 x 500 x 1800; other sizes and lengths on demand

All values are average values / typical ranges

Figure S26. Technical specification of the graphite used.

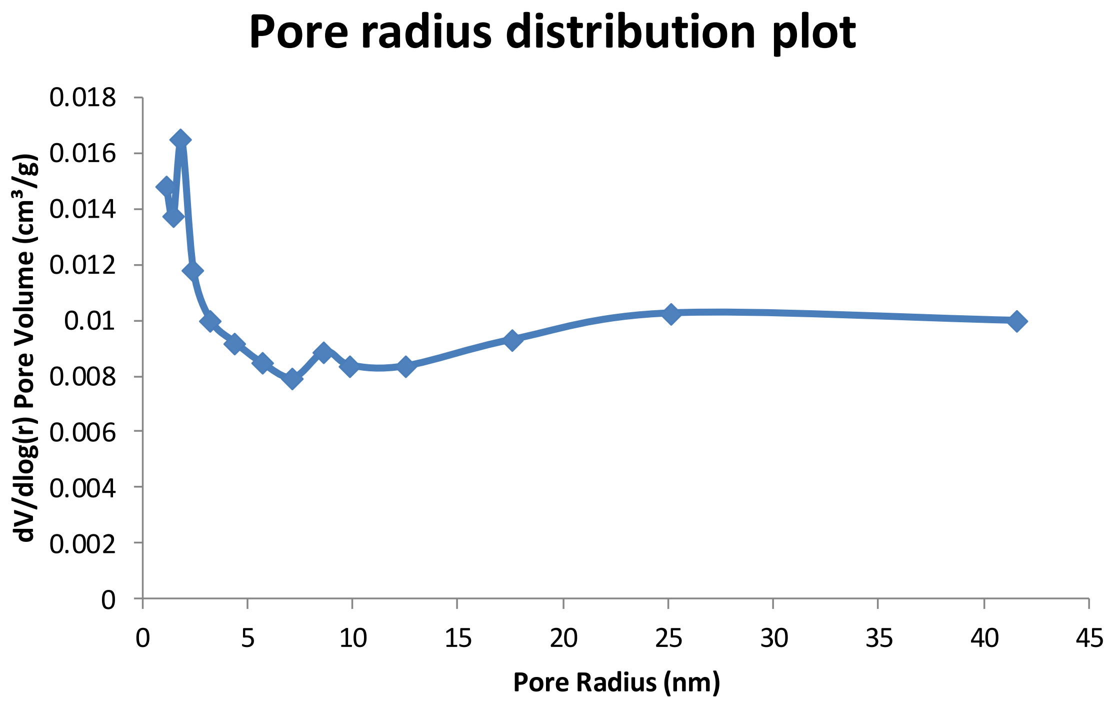

Figure S27. Pore size distribution plot of the carbon used in the graphite liner.

Electrode calibration

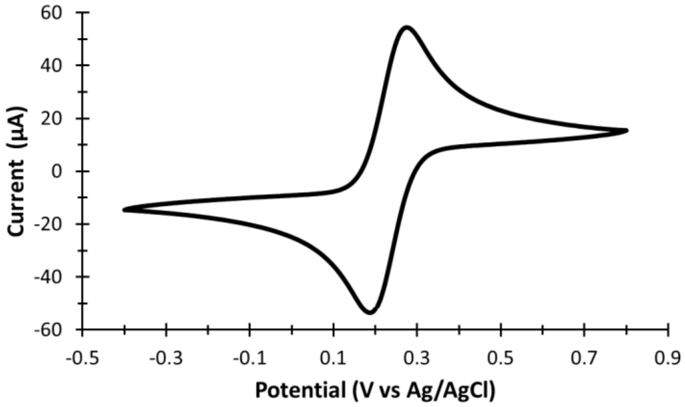

Figure 28 Example cyclic voltammogram of 10 mM ferricyanide in 0.1 M pH7 phosphate buffer between -0.4 and 0.8 V vs Ag/AgCl for the calibration relative to SHE. Experimental conditions: starting potential: 0.2 V vs Ag/AgCl; scan rate: 50 mV s^{-1}; current range: 1 mA to 1 nA; total number of scans: 8; equilibration time: 10 s.

Table S4. Calculating the reference electrode conversion factor.

Run	$E_{p,a}$ (V vs Ag/AgCl)	$E_{p,c}$ (V vs Ag/AgCl)	$E_{1/2}$ (V vs Ag/AgCl)	Reported $E_{1/2}$ (V vs SHE)[7]	Conversion factor for V vs Ag/AgCl to V vs SHE (V)
1	0.275	0.185	0.230		
2	0.285	0.180	0.233	0.425	0.194
3	0.285	0.175	0.230		
		**Average**	**0.231 ± 0.0014**		

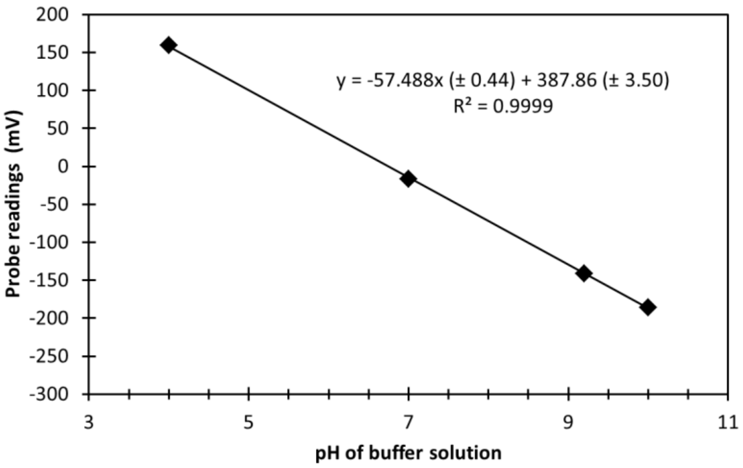

Figure S29 Example pH calibration curve using four reference buffer solutions from Fisher-Scientific, pH 4 (potassium acid phthalate), 7 (phosphate), 9.2 (borate) and 10 (potassium carbonate).

Seawater collection and analysis

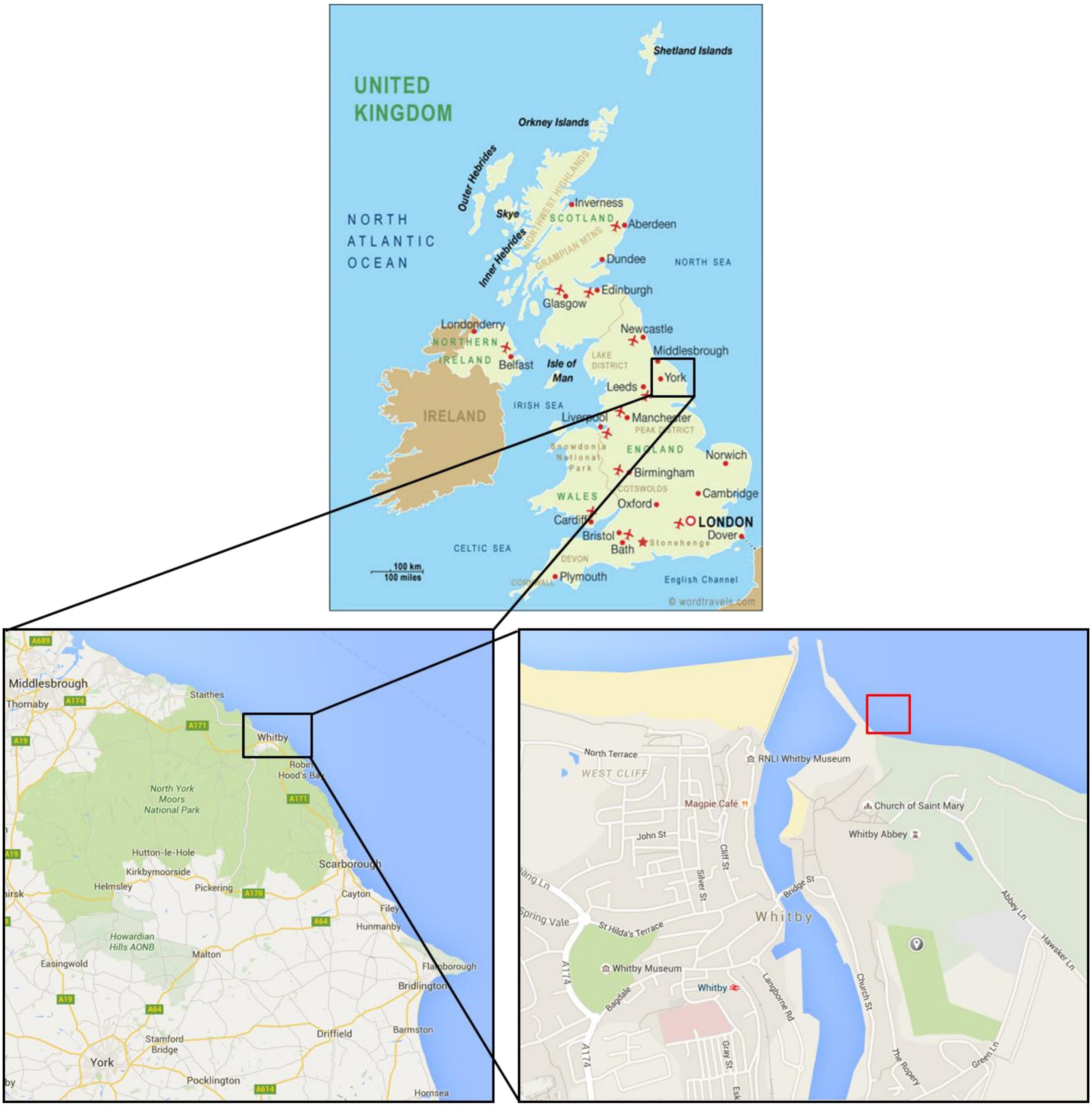

Figure S30. Maps showing location from which seawater was collected.

Table S5. Elemental analysis of seawater via ICP-MS. Only detected elements are shown.

Na / %	Mg / %	K / %	Ca / %	S / %
1.23 (± 5.1%)	0.15 (± 0.5%)	0.05 (± 0.4%)	0.05 (± 3.9%)	0.15 (± 6.6%)

References

[1] A. Belsky, M. Hellenbrandt, V. L. Karen, P. Luksch, *Acta Crystallogr. Sect. B* **2002**, *58*, 364–369.
[2] G. E. Bacon, N. A. Curry, *Acta Crystallogr.* **1956**, *9*, 82–85.
[3] G. Chapuis, M. Dusek, M. Meyer, V. Petricek, *Acta Crystallogr. Sect. B* **2003**, *59*, 337–352.
[4] B. Bureau, G. Silly, J. Y. Buzaréa, C. Legein, D. Massiot, *Solid State Nucl. Magn. Reson.* **1999**, *14*, 181-190.
[5] E. Deltombe, M. Pourbaix, *Corrosion* **1958**, *14*, 16–20.
[6] G. Bergerhoff, I. D. Brown, eds., *Inorganic crystal structure database*, International Union of Crystallography, Chester, England, **1987**.
[7] J. E. O'Reilly, *Biochim. Biophys. Acta, Bioenerg.* **1973**, *292*, 509–515.
